# Regulation of transcriptional homeostasis by DNA methylation upon genome duplication in pak choi

**DOI:** 10.1186/s43897-025-00145-3

**Published:** 2025-04-05

**Authors:** Min Ma, Yuanda Wang, Zhenfei Sun, Ranze Zhao, Honghua Li, Xiaoxuan Li, Hongfang Zhu, Xuedong Yang, Changwei Zhang, Yuda Fang

**Affiliations:** 1 Joint Center for Single Cell Biology, School of Agriculture and Biology, Shanghai Jiao Tong University, Shanghai, 200240 China; 2 Shanghai Key Laboratory of Facility Horticulture Technology, Shanghai Academy of Agricultural Sciences, Shanghai, 201403 China; 3 College of Horticulture, Nanjing Agricultural University, Nanjing, 210095 China

**Keywords:** Autopolyploid, Pak choi, Transcriptional regulation, Transposable element, DNA methylation

## Abstract

Polyploidy occurs frequently in plants and is an important force in plant evolution and crop breeding. New polyploids face various challenges due to genome duplication and subsequent changes in epigenetic modifications, nucleus/cell size and gene expression. How polyploids produce evolutionary novelty remains to be understood. In this study, a transcriptome comparison between 21-day-old diploid and autotetraploid pak choi seedlings revealed that there are few differentially expressed genes (DEGs), with a greater proportion of DEGs downregulated in response to genome duplication. Genome-wide DNA methylation analysis indicated that the level of DNA methylation is obviously increased, especially in transposable elements (TEs) and 1 kb flanking regions, upon genome doubling. The differentially methylated regions between diploid and autotetraploid pak choi were related to 12,857 differentially hypermethylated genes and 8,451 hypomethylated genes, and the DEGs were negatively correlated with the differential methylation in the regions across the DEGs. Notably, TE methylation increases significantly in regions flanking neighboring non-DEGs rather than those flanking DEGs. These results shed light on the role of DNA methylation in the transcriptional regulation of genes in polyploids and the mechanism of coping with “genome shock” due to genome doubling in cruciferous plants.

**Supplementary Information:**

The online version contains supplementary material available at 10.1186/s43897-025-00145-3.

## Core

In autotetraploid pak choi, non-DEGs are characterized by elevated DNA methylation levels of TEs flanking these non-DEGs. In contrast, the transcription level of DEGs overlapping with DMGs was negatively correlated with the DNA methylation level across these DEGs.

## Gene and accession numbers

The RNA-seq and WGBS data generated in this study have been deposited in the National Center for Biotechnology Information (NCBI) under accession number PRJNA1017384.

## Introduction

Polyploidy, or whole‒genome duplication (WGD), is the doubling of the number of intact chromosomes in somatic cells to three or more groups (Frawley et al. 2015). Seventy percent of angiosperms have experienced one or more genome doubling events (Masterson and Masterson 1994). For example, the*Arabidopsis thaliana*genome has undergone at least four large-scale genome duplication events, resulting in the genome being covered by a large number of segmental duplications (Vision et al. 2000). Polyploids formed via duplication of a single genome are called autopolyploids, while allopolyploids refer to individuals of hybrid origin apart from genome duplication. In addition, there are also autoallopolyploids that combine the characteristics of both autopolyploids and allopolyploids (Stebbins 1947; Jackson and Jackson 1982). Recent studies have shown that both autopolyploids and allopolyploids are important evolutionary forces in plants (Doyle et al. 2019; Jiao et al. 2011; Barker et al. 2016).

Reported first in *O. Lamarckiana*in 1907 (Lutz and Lutz 1907), polyploids often show changes in plant morphology, delayed growth, late flowering or a longer growth cycle, which makes them well suited for ornamental breeding (Stebbins 1947), (Corneillie et al. 2019). Compared with their diploid progenitor, polyploid plants frequently exhibit increased cell size and larger organs, known as the “gigas” effect, resulting in higher yields of fruits and vegetables (Stebbins 1950; Levin and Levin 1983). In addition, the redundancy caused by the doubling of the genome results in the duplicated genome having the characteristics of “buffering” and high heterozygosity (Levin 2002; Soltis PS and Soltis DE 2000; Comai and Comai 2005). It was proposed that polyploid species have greater adaptability and increased tolerance to various environmental changes (Diallo et al. 2016; Van de Peer et al. 2009). Many of the organisms that produced polyploids survived, but many other plants and animals perished forever in the Cretaceous–Tertiary extinction event (Fawcett et al. 2009; Vanneste et al. 2014). A study of polyploid plants in the Arctic revealed that polyploids in newly deglaciated areas invade more successfully than diploids do, possibly because a fixed heterozygous genome buffers inbreeding and genetic drift due to dramatic climate change (Brochmann et al. 2004). Given that polyploid plants exhibit extreme phenotypes and traits that are beneficial to agriculture, plant breeders have been working to develop synthetic polyploid species with high stress tolerance and productivity (Santantonio et al. 2019).

The genomic regulation in polyploids is genome-wide nonadditive and nonrandom (Leitch and Leitch 2008). There was less variation in gene transcription levels during genome doubling in*Arabidopsis*(Zhang et al. 2019). In polyploids, there are complex feedback patterns among genome size, cell size, transcription, the cell cycle, metabolism, and other processes, and these multisystemic changes allow the formation of new regulatory pathways (Scarrow et al. 2021). A series of*Arabidopsis* tetraploids containing different dosages of *Arabidopsis thaliana* and *Arabidopsis arenosa*genomes were generated for studying genome-wide correlation between allelic transcription and dosage change (Shi et al. 2015). It has been shown that the dosage-dependent genes are involved in ancient biochemical pathways not specific to plants, whereas many dosage-independent genes in plant-specific pathways (Shi et al. 2015). Apart from gene dosage changes, genome doubling is not involved in DNA sequence changes, resulting in epigenetically induced genomic regulation (Seoighe and Gehring 2004; Adams et al. 2005). In*Arabidopsis*autotetraploids, ploidy-induced genomic changes are closely related to epigenetic inheritance (Del Pozo and Ramirez-Parra 2015). Phenotypic changes caused by polyploidization result predominantly from changes in epigenetic modifications (Feng et al. 2010; Song Q and Chen ZJ 2015; Aversano et al. 2013; Feng S, Jacobsen SE 2011; Li 2021; Holliday 1994).

Epigenetic markers and related mechanisms are often ancient and conserved, including DNA methylation, histone variation and modification, nucleosome localization, noncoding RNAs and three-dimensional organization of the genome (Skvortsova et al. 2018). The predominant form of DNA methylation is the addition of a methyl group to the fifth carbon of a cytosine in the eukaryotic genome to produce 5mC (Luo et al. 2018). DNA methylation is generally associated with transposon silencing, imprinting, development and environmental responses and is dynamically regulated by de novo methylation, methylation maintenance and demethylation activities (Pikaard et al. 2014; Schübeler 2015; Xie et al. 2024; Jiang et al. 2024). In mammals, DNA methylation is predominantly found at CpG dinucleotides, with the majority of these sites clustered within CpG islands (Esteller and Esteller 2008). In contrast, plants exhibit DNA methylation across three sequence contexts: CG, CHG, and CHH (H represents A, T or C) (Zhang et al. 2006; Zhang et al. 2018). The level of 5mC in plant DNA is notably high, with variation observed across different sequence contexts. 5mCG tends to have the highest level of DNA methylation across the entire genome, varying by up to threefold among species, from 30.5% in*Arabidopsis* to 92.5% in sugar beet (*Beta vulgaris*). The interspecies variation in 5mCHG levels was approximately eightfold, ranging from 9.3% in salt mustard (*Eutrema salsugineum*) to 81.2% in sugar beet. The 5mCHH level is generally the lowest but shows the greatest variation, with a difference of approximately 16 times. The highest level of mCHH was found in sugar beet, reaching 18.8%. This percentage is unusually high, as 85% of the species had mCHH levels less than 10%, and half of the species had mCHH values less than 5%. The lowest level of mCHH was found in *Vitis vinifera*(1.1%). Notably, sugar beet has the highest levels of DNA methylation across all contexts, with a particularly high mCHH level (Niederhuth et al. 2016).

Different DNA methyltransferases are responsible for different contexts of methylation. CG, CHG and CHH methylation are maintained by METHYLTRANSFERASE 1 (MET1), CHROMOMETHYLASE 3 (CMT3), and DOMAINS REARRANGED METHYLASE 2 (DRM2) or CMT2, respectively (Zhang et al. 2018; Matzke et al. 2014). In plants, the RNA-directed DNA methylation (RdDM) pathway is important for de novo DNA methylation (Matzke et al. 2014; Zhang et al. 2011).

Upon genome doubling, the plant genome undergoes a wide range of chromatin state changes, including alterations in DNA methylation and histone modification. Subtle changes in CG and CHG DNA methylation were detected in two synthetic potato autotetraploids (Aversano et al. 2013). Hypermethylated transposable elements (TEs) buffer the effects of chromatin structure changes on gene transcription during genome doubling in A/B switching regions and TAD boundary genes in rice autotetraploids (Sun et al. 2022). The CHH methylation of genes associated with fatty acid and JA biosynthesis contributes to cold tolerance in autotetraploid*Poncirus trifoliata*(Wang et al. 2022). Investigating the epigenetic regulatory mechanisms involved in polyploidy, particularly genome stability, is important for the development of novel crop varieties and the enhancement of their quality. Pak choi (*Brassica rapa* ssp*. chinensis*) is a popular vegetable with a long history of cultivation and rich germplasm resources in China (Hou 2020; Zhang et al. 2020). Compared with its diploid progenitor, autopolyploid pak choi normally flowers later and has larger organs and thicker leaves, resulting in better performance and productivity at harvest (Zhang et al. 2020; Han et al. 2014). As the seedling stage is the key bottleneck period in the process of plant growth and development (Harper 1977; Leck 2008), we investigated the level and distribution of DNA methylation and its correlation with the gene expression of 21-day-old pak choi upon genome doubling. Our results revealed genomic maps of DNA modifications in accordance with gene transcription and key regulatory mechanisms in autotetraploid pak choi.

## Results

### Phenotypic and transcriptomic changes upon genome doubling of pak choi

**Fig. 1:**
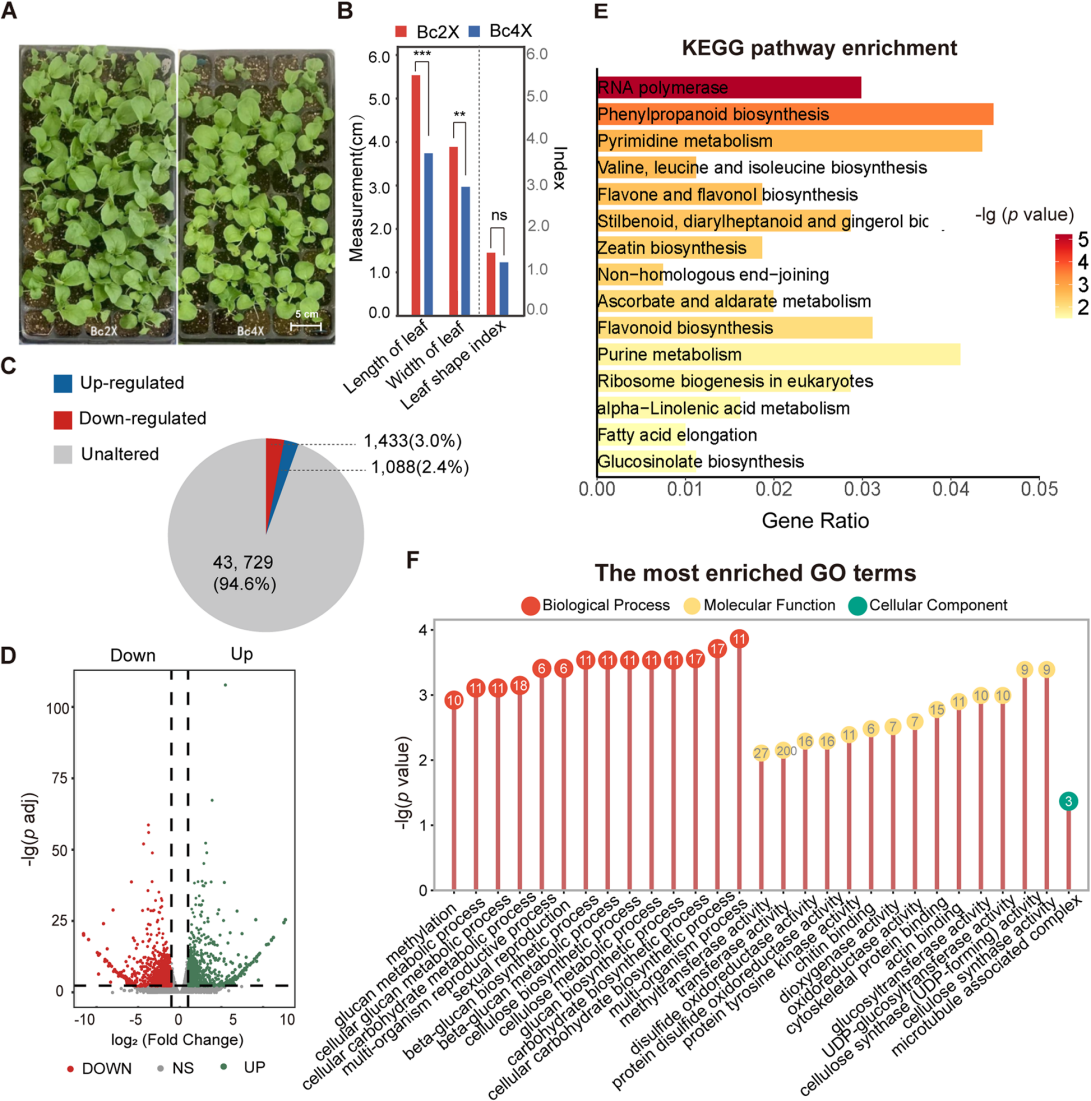
Characterization of DEGs between Bc2X and Bc4X. **A** Visual phenotypes of 21-day-old Bc2X (left) and Bc4X (right) seedlings. **B** Comparison of leaf length, width, and the leaf shape index between 21-day-old Bc2X and Bc4X seedlings. Statistical analysis was performed with two-tailed Student’s *t* tests; *P* values: “**” < 0.01, “***” < 0.001. “cm” = centimeter. **C** Pie chart showing the proportions of upregulated genes (blue) and downregulated genes (red) relative to total genes between Bc2X and Bc4X (fold change >  = 2.00 and adjusted *p* value <  = 0.01). **D** Volcano plot showing the distribution of fold changes and *p* values for the upregulated genes (right) and downregulated genes (left). **E** KEGG pathway enrichment analysis of DEGs between Bc2X and Bc4X. **F** GO analysis of DEGs between Bc2X and Bc4X

To study transcriptional regulation during the genome doubling of pak choi, we performed RNA sequencing (RNA-seq) to quantify the transcripts in leaves of diploid (Bc2X) and autotetraploid (Bc4X) 21-day-old seedlings whose full true leaves were shorter and rounder in Bc4X than in Bc2X (Fig. 1A-B). Approximately 5.02 ~ 7.16 Gb of quality-filtered reads were obtained for each of the three replicate libraries with Q30 score > 91.4% (Table S1). Hierarchical clustering, cluster expression heatmaps, and PCA confirmed the reliability of the biological replicates, as well as the difference in the RNA-seq results between Bc2X and Bc4X (Figure S1A-C). The transcript levels of several randomly selected genes from Bc2X and Bc4X were further validated via quantitative real-time polymerase chain reaction (qRT‒PCR), which confirmed the reliability and repeatability of the sequencing results (Figure S1D). In addition, the overall transcript levels of the Bc4X genes were not significantly different from those of Bc2X, indicating that the Bc4X genes are transcriptionally regulated in a dose-independent manner (Figure S1E, Table S2). Among all the detected genes, 2,521 differentially expressed genes (DEGs) were identified, with 1,088 upregulated and 1,433 downregulated in Bc4X compared with Bc2X (fold change >  = 2.00 and adjusted *p* value <  = 0.01) (Fig. 1C-D). The low proportion of DEGs among the total genes annotated from the *B. rapa* reference genome (2,521/46,250 = 5.45%) and a genome-wide transcription heatmap of Bc2X and Bc4X (Figure S2) indicated that the transcriptome was not drastically altered upon genome doubling.

### DNA methylation changes upon genome duplication in pak choi

Kyoto Encyclopedia of Genes and Genomes (KEGG) analysis revealed enrichment of DEGs related to RNA polymerase (*p* value = 5.59e^−6^), phenylpropanoid biosynthesis (*p* value = 0.0003), and pyrimidine/purine metabolism (*p* value = 0.0002) (Fig. 1E). A comparison of the transcript levels of Pol I-Pol III-specific subunit genes between Bc2X and Bc4X revealed that only one Pol II-specific subunit, NRPB4 (DNA-DIRECTED POL II SUBUNIT 4), exhibited differential expression (Table S3). In addition to Pol I-III, two plant-specific RNA polymerases, Pol IV and Pol V, which are related to the RdDM pathway, were also examined (Xie et al. 2023). Among the Pol IV- and Pol V-specific subunits, five genes, including*BcNRPD2* (*DNA-DIRECTED POL IV SUBUNIT 2*), *BcNRPD4*, *BcNRPE5* (*DNA-DIRECTED POL V SUBUNIT 5*), *BcDRD1* (*DEFECTIVE IN RNADIRECTED DNA METHYLATION 1*) and *BcDMS3* (*DEFECTIVE IN MERISTEM SILENCING 3*), exhibited significant differences in transcription (Figure S3A-B, *p* value < 0.05). Moreover, Gene Ontology (GO) analysis revealed that DEGs were enriched in the biological process terms of methylation (*p* value = 0.0012) and the molecular function terms of methyltransferase activity (*p* value = 0.0078) (Fig. 1F), indicating that the transcription levels of some DNA methylation-related genes may change upon genome doubling in pak choi. We then investigated the transcription levels of DNA methyltransferase and demethylase genes in Bc4X. Using BLASTP and phylogenetic trees with the maximum likelihood method, we identified seven methyltransferases and four demethylase homologs (Figure S4A, Table S4). Among these seven genes, the transcription levels of four genes decreased (Figure S4B-C) with increasing expression of the demethylases *BcROS1* and *BcDME* (TMM > 3, *p* value < 0.05). Subsequently, key genes related to the RdDM pathway were identified (Figure S3A, Table S4). Five genes (*BcNRPD4*, *BcNRPE5*, *BcDRD1*, *BcHDA6* (*HISTONE DEACETYLASE6*) and *BcDMS3*) were upregulated, whereas eleven genes (*BcNRPD2*, *BcDCL3* (*DICER-LIKE PROTEIN 3*), *BcDCL4*, *BcHEN1-1* (*HUA1 ENHANCER1*), *BcIDN2-1* (*INVOLVED IN *DE NOVO* 2*), *BcSUVR2-2* (*SUPPRESSOR OF VARIEGATION RELATED 2*), *BcLDL1-2* (*LYSINE-SPECIFIC DEMETHYLASE 1-LIKE 1*), *BcJMJ14* (*JUMONJI14*), *BcDDM1-2* (*DECREASED DNA METHYLATION 1*), *BcMOM1-1* (*MORPHEUS MOLECULE 1*) and *BcMOM1-2*) were downregulated (Figure S3B, *p* value < 0.05). These results suggest that DNA methylation might be altered during genome duplication in pak choi.

**Fig. 2:**
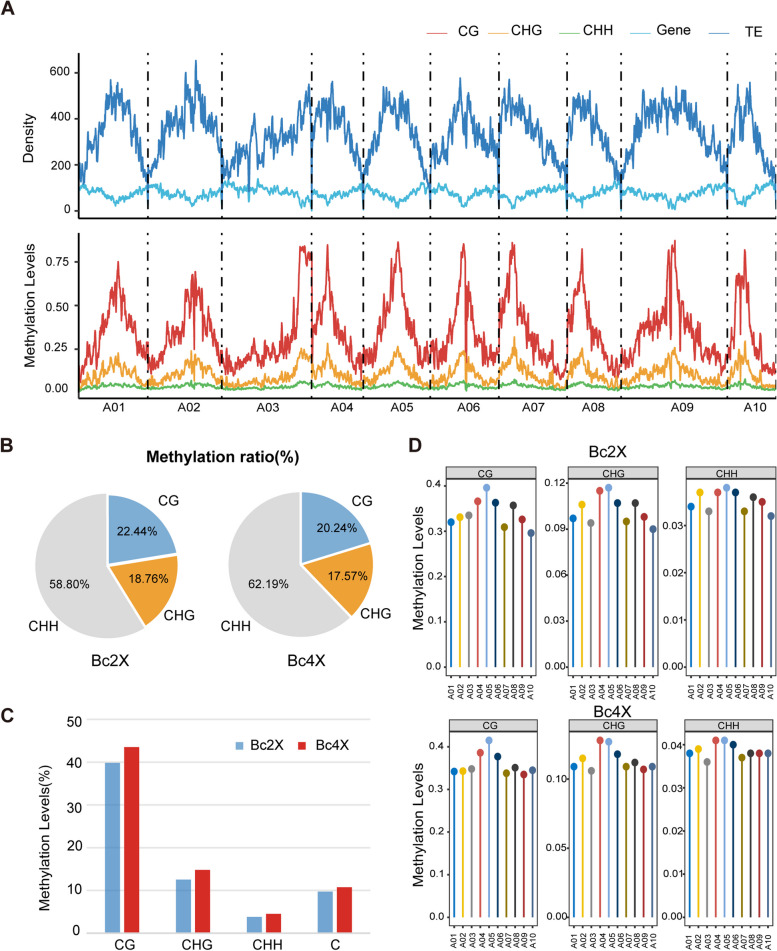
Genome-wide DNA methylation comparison between Bc2X and Bc4X. **A** The plots showing DNA methylation in three sequence contexts (CG, CHG, CHH) of Bc2X and the densities of genes and TEs across chromosomes. **B** Pie charts showing the proportional distribution of methylated C sites in each sequence context for Bc2X (left) and Bc4X (right). **C** DNA methylation levels in three sequence contexts (CG, CHG, and CHH) for Bc2X and Bc4X. **D** Plots showing the distribution of DNA methylation on different chromosomes for three sequence contexts (CG, CHG, CHH) of Bc2X (upper panel) and Bc4X (lower panel)

Whole-genome bisulfite sequencing (WGBS) was then performed on the leaves of 21-day-old Bc2X and Bc4X seedlings, with two biological replicates. The amount of high-quality data after quality control was sufficient (average Q20 > 95.10%, Q30 > 89.40%) (Table S5). From a genome-wide perspective, Bc2X and Bc4X have similar DNA methylation patterns with higher methylation levels at CG and CHG sites containing dense TEs, and CHH methylation displayed gentle distribution (Fig. 2A, Figure S5). For the proportion of 5mC in the CHH context, 58.80% and 62.19% are in Bc2X and Bc4X; for the CG context, 22.44% and 20.24% are in Bc2X and Bc4X; for the CHG context, 18.76% and 17.57% are in Bc2X and Bc4X, respectively (Fig. 2B). These results indicated that the proportion of methylated C sites in Bc4X decreases in the CG and CHG contexts but increases in the CHH context compared with Bc2X. In the CG context, the average methylation levels of cytosine in Bc2X and Bc4X were 39.83% and 43.51%; in the CHG context, they were 12.53% and 14.78%; and in the CHH context, they were 3.80% and 4.53%, respectively (Fig. 2C), suggesting that the DNA methylation levels of Bc4X are higher than those of Bc2X in all three C contexts. These results indicate that the DNA methylation level in different contexts increases markedly after genome doubling, with the CHH context showing the greatest increase. Furthermore, the CG, CHG, and CHH sequence contexts of the methylation levels on all chromosomes (A01-A10) were observed (Fig. 2D). The results suggested that the highest methylation level is located on A05 in both Bc2X and Bc4X, except for the CHG context in Bc4X, which has the highest methylation level on A04. The lowest DNA methylation level in Bc2X was on A10 for all three C contexts, whereas in Bc4X, the lowest DNA methylation level was found on A09 in the CG context and on A03 in the CHG and CHH contexts.

### DNA methylation changes in autotetraploid pak choi are related to various genomic elements and subtrinucleotide contexts

**Fig. 3:**
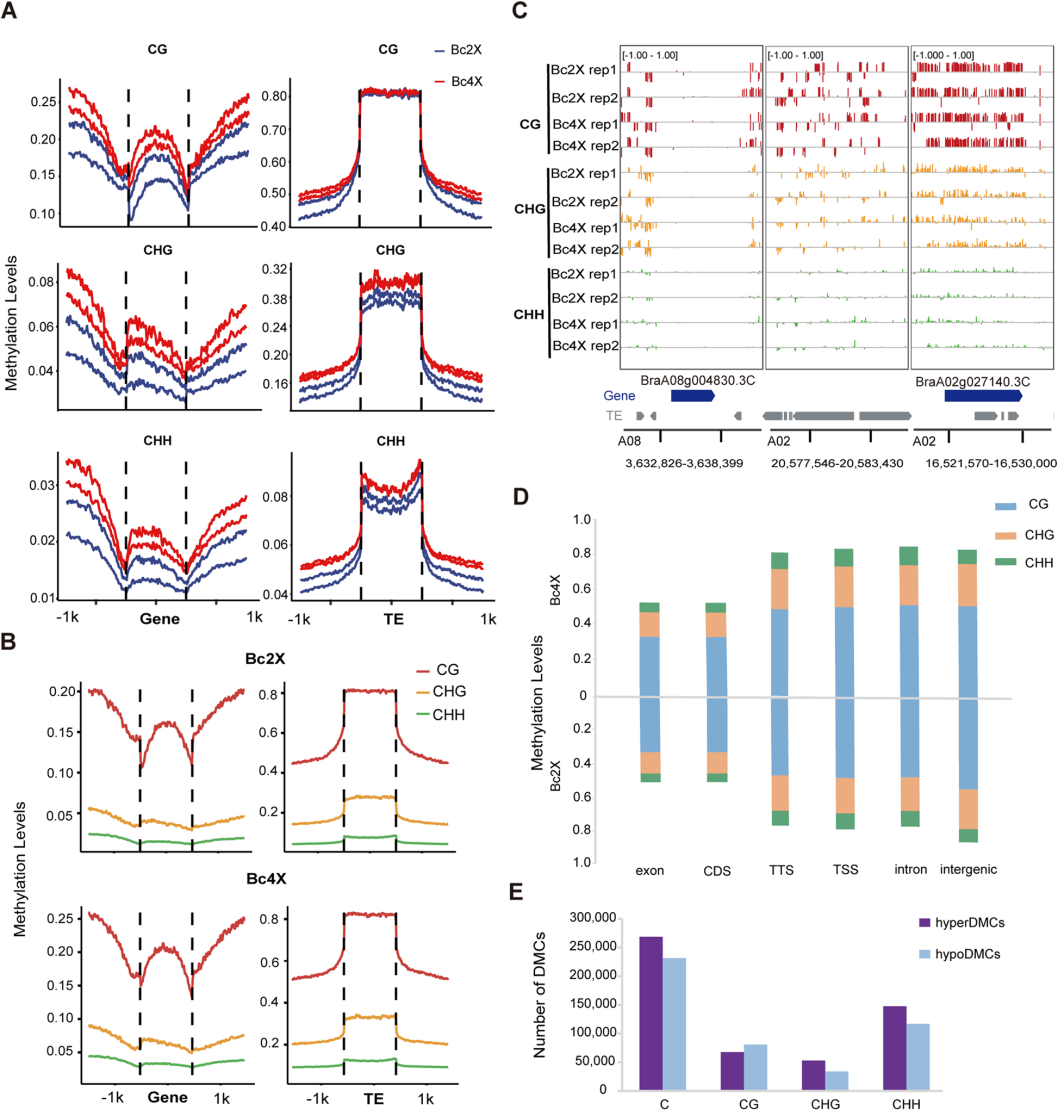
Differential DNA methylation profiles of Bc2X and Bc4X. **A** Distribution of the average methylation levels of Bc2X (blue) and Bc4X (red) across gene bodies and TEs and 1 kb flanking regions in the CG (upper panel), CHG (middle panel) and CHH (lower panel) contexts. Lines of the same color represent two biological replicates. **B** DNA methylation distribution of Bc2X (upper panel) and Bc4X (lower panel) in the three sequence contexts (CG, CHG, CHH) in genes, TEs and 1 kb flanking regions. Lines of the same color represent CG, CHG and CHH. **C** IGV plot of the DNA methylation levels of two genes (*BraA08g004830.3C* A08:3,634,826–3,636,399, *BraA02g027140.3C* A02:16,52,3570–16,528,000) and one TE (A02:20,577,546–20,583,430). Lines of the same color represent CG, CHG and CHH. **D** Histogram of methylation levels in different genome elements (exons and introns, intergenic regions and CDSs, TSSs and TTSs) for Bc2X (lower panel) and Bc4X (upper panel); three different colors represent CG, CHG and CHH, respectively. **E** Numbers of hyperDMCs and hypomethylated-DMCs (hypoDMCs) between Bc2X and Bc4X are shown for the CG, CHG, and CHH sequence contexts

We then compared the DNA methylation levels among different genomic elements of Bc2X and Bc4X. Methylation analysis of gene bodies/TEs and 1 kb flanking regions revealed that the methylation levels of Bc4X are always higher in all three C sequence contexts than those of Bc2X (Fig. 3A). The methylation distribution patterns across genes and TEs are largely similar between Bc2X and Bc4X, with higher DNA methylation levels in TEs than in genes. In addition, in both Bc2X and Bc4X, CG methylation levels in genes and TEs are the highest, whereas CHH methylation levels are the lowest among the three sequence contexts (Fig. 3B). By visualization with the Integrative Genomics Viewer (IGV), we observed that the methylation levels in the 1 kb regions flanking the gene bodies were greater than those in the gene bodies. In contrast, the methylation levels in the 1 kb regions flanking TEs were lower than those in the TE bodies (Fig. 3C).

To clarify the distribution of methylation in the genome, we divided the whole genome into exons/introns, intergenic regions, CDSs, transcription start sites (TTSs) and transcription termination sites (TSSs). We found that the methylation levels of Bc2X and Bc4X were greater in intergenic regions, introns, TTSs and TSSs than in CDSs (Fig. 3D). In addition, we identified a total of 499,963 differentially methylated cytosines (DMCs) in Bc4X compared with Bc2X. Among these DMCs, 53.7% are hypermethylated DMCs (hyperDMCs), and 52.9% of these hyperDMCs occur in the CHH context, suggesting that CHH has the widest range of methylation changes upon genome duplication (Fig. 3E).

**Fig. 4:**
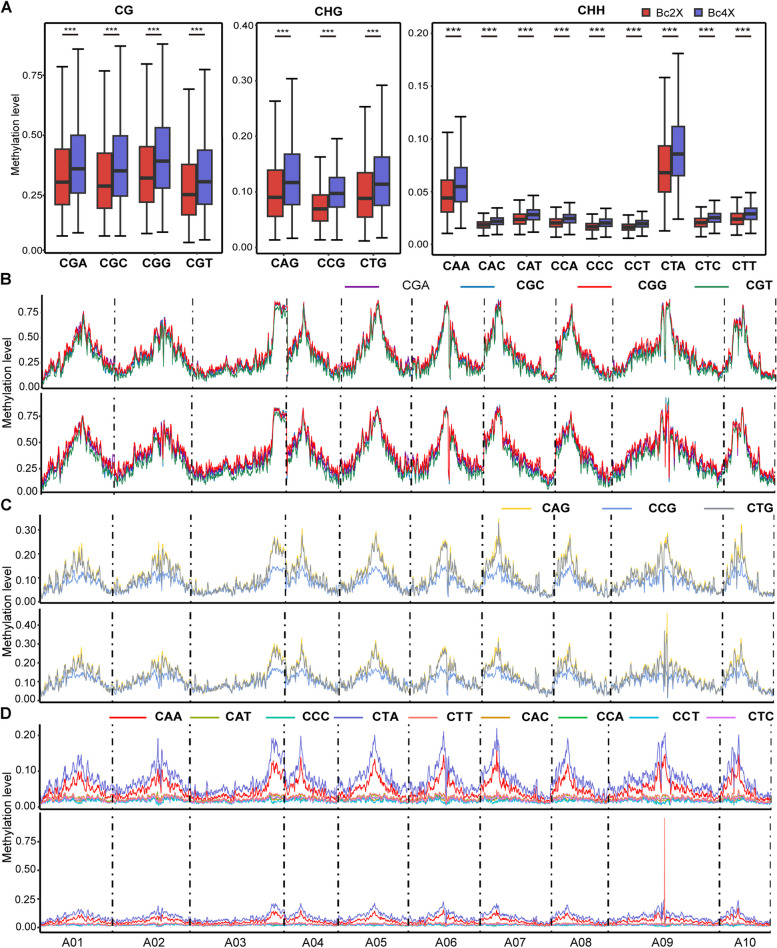
Genome-wide DNA methylation distributions across different subcontexts in the CG, CHG and CHH contexts. **A** Boxplots showing the methylation levels of each subcontext under CG, CHG and CHH contexts across chromosomes per 500 kb bin in Bc2X and Bc4X. Statistical analysis was performed with two-tailed Wilcoxon tests. *P* value: “***” < 0.001. **B** Plots showing the methylation levels of CG subcontexts across chromosomes per 500 kb bin (upper panel is Bc2X, lower panel is Bc4X). **C** Plots showing the methylation levels of CHG subcontexts across chromosomes per 500 kb bin (upper panel is Bc2X, lower panel is Bc4X). **D** Plots showing the methylation levels of CHH subcontexts across chromosomes per 500 kb bin (upper panel is Bc2X, lower panel is Bc4X)

In *Arabidopsis*, cytosines in different subcontexts can be differentially methylated (Lister et al. 2008). To explore the dynamic changes in DNA methylation in autopolyploids, we analyzed the DNA methylation levels of Bc2X and Bc4X in trinucleotide subcontexts. First, we calculated the density of different trinucleotides in the*B. rapa* genome. For the CG context, CGA has the highest density, whereas CGC has the lowest density (Figure S5A). For their methylation levels, the highest methylation level under all four subcontexts was CGG, and the lowest was CGT. For the CHG context, the density of the CAG subcontexts is the highest, and that of the CCG subcontexts is the lowest, while the CTG methylation level is similar to that of the CAG subcontexts. For the CHH context, the CAA has the highest density, whereas the CCC has the lowest density (Fig. 4A). The subcontext with the highest methylation level under nine different subcontexts in Bc2X and Bc4X was CTA, and the lowest was CCC. In addition, Bc4X has higher DNA methylation levels in all nine different subcontexts than Bc2X does. In different subcontexts, DNA methylation has a bias in the CGG subcontext of the CG and CTA subcontext of the CHH. Through global analysis of DNA methylation levels and density distributions in trinucleotide contexts, we observed that the DNA methylation patterns of Bc2X and Bc4X are similar under CG and CHG, whereas Bc4X has the highest CTT subcontext under CHH on chromosome A09 (Fig. 4B-D, Figure S5B).

### The differentially expressed genes in autotetraploid pak choi overlap significantly with differentially methylated genes

**Fig. 5:**
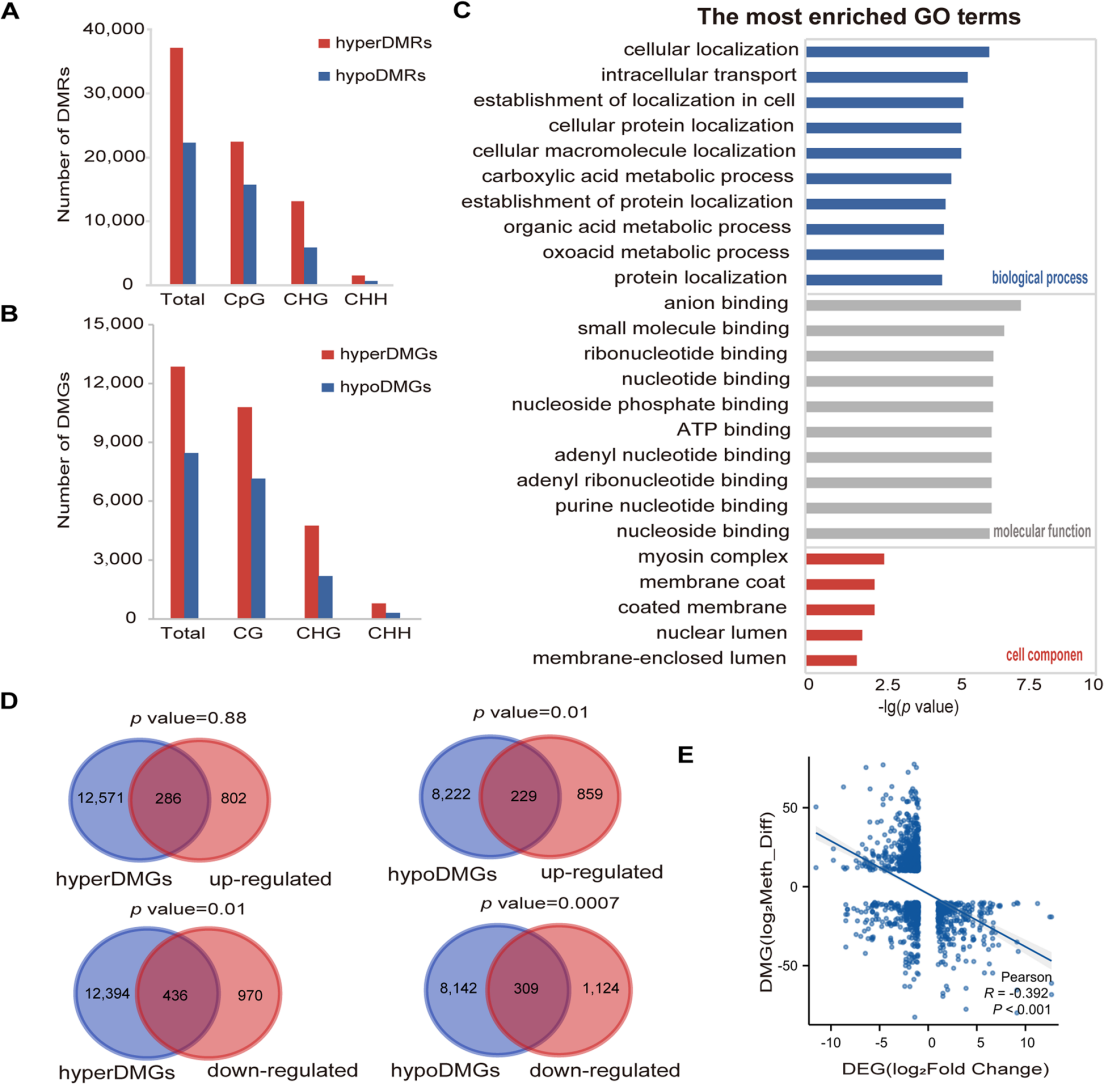
Functional prediction of DMGs and enrichment with DEGs. **A** Numbers of DMRs between Bc2X and Bc4X for the CG, CHG, and CHH sequence contexts. Red bars represent hyperDMRs, and blue bars represent hypoDMRs. **B** Numbers of DMGs between Bc2X and Bc4X for the CG, CHG, and CHH sequence contexts. **C** GO analysis of DMGs between Bc2X and Bc4X. **D** Venn diagram showing the overlaps between the DEGs (up/downregulated) and the DMGs (hyper/hypoDMGs). Significance was examined via the hypergeometric test. **E** Scatter plots showing the correlations between methylation and transcription differences of the significantly enriched genes in** D**. Statistical analysis was performed with two-tailed Student’s *t* tests

To explore the relationship between DNA methylation and gene expression, we first identified and compared the differentially methylated regions (DMRs) and DMR-related genes (DMGs) between Bc2X and Bc4X (Fig. 5A-B). The results indicated that the number of differentially hypermethylated regions (hyperDMRs) in the CG, CHG and CHH contexts was greater than that of the differentially hypomethylated regions (hypoDMRs), and the number of DMRs was greatest in the CG context and lowest in the CHH context (Fig. 5A). The higher proportion of hyperDMRs is consistent with the increased overall methylation level of Bc4X. There are 12,857 differentially hypermethylated genes (hyperDMGs) and 8,451 differentially hypomethylated genes (hypoDMGs). GO analysis revealed that the DMGs were enriched mainly in the biological processes of cellular localization, the molecular functions of anion binding, and the cellular components of the myosin complex (Fig. 5C). We then conducted enrichment analysis between hyperDMGs/hypoDMGs and up- or downregulated DEGs. We found that the upregulated DEGs overlapped significantly with hypoDMGs but not with hyperDMGs (Fig. 5D), whereas the downregulated DEGs significantly overlapped with both hyperDMGs and hypoDMGs. We further conducted enrichment analysis between the downregulated DEGs and hypoDMGs with altered DNA methylation of gene bodies or 1 kb flanking regions, as the methylation of the gene body has been shown to be positively correlated with gene transcription (Yang et al. 2014). The results revealed that the downregulated DEGs were significantly enriched with hypoDMGs in gene bodies but not in the flanking regions (Figure S7A). In contrast, the non-DEGs did not significantly overlap with hyperDMGs or hypoDMGs (Figure S7B).

The transcription level and DNA methylation across DEGs that significantly overlapped with DMGs were associated, and the results indicated that they were negatively correlated, especially CG methylation (Fig. 5E, Figure S7C). GO analysis revealed that the genes significantly overlapping between DEGs and DMGs in the CG context were enriched mainly in the biological processes of methylation, the molecular functions of cellulose synthase activity, and the cellular components of the extrinsic component of the membrane (Figure S7D).

We then investigated the relationship between differential methylation and differential transcription via the inhibition of DNA methylation. Bc2X and Bc4X were treated with 5-Aza (5-Azacytidine), a DNA methylation inhibitor, and the DNA methylation status in 1 kb regions flanking the hyperDMGs after 5-Aza treatment was monitored by Chop-PCR (methylation-sensitive enzyme digestion followed by PCR), a targeted DNA methylation detection technique (Dasgupta et al. 2019) (Figure S8). The Chop‒PCR results indicated that the DNA methylation levels of*BcPIN4* (*PIN-FORMED 4*), *BcLINC1 (LITTLE NUCLEI1)* and *BcBRZ1* (*BRASSINAZOLE RESISTANT1*) were decreased upon 5-Aza treatment in both Bc2X and Bc4X (Figure S8). The qRT‒PCR results revealed that the relative gene transcription levels (Bc4X vs. Bc2X) of these downregulated genes significantly increased after the DNA methylation of these genes was inhibited (Figure S8), indicating a negative correlation between the transcription level of these hyperDMGs and the DNA methylation level in the 1 kb regions flanking these genes.

### Differential TE methylation between Bc2X and Bc4X affects the transcription of TE-neighboring genes

**Fig. 6:**
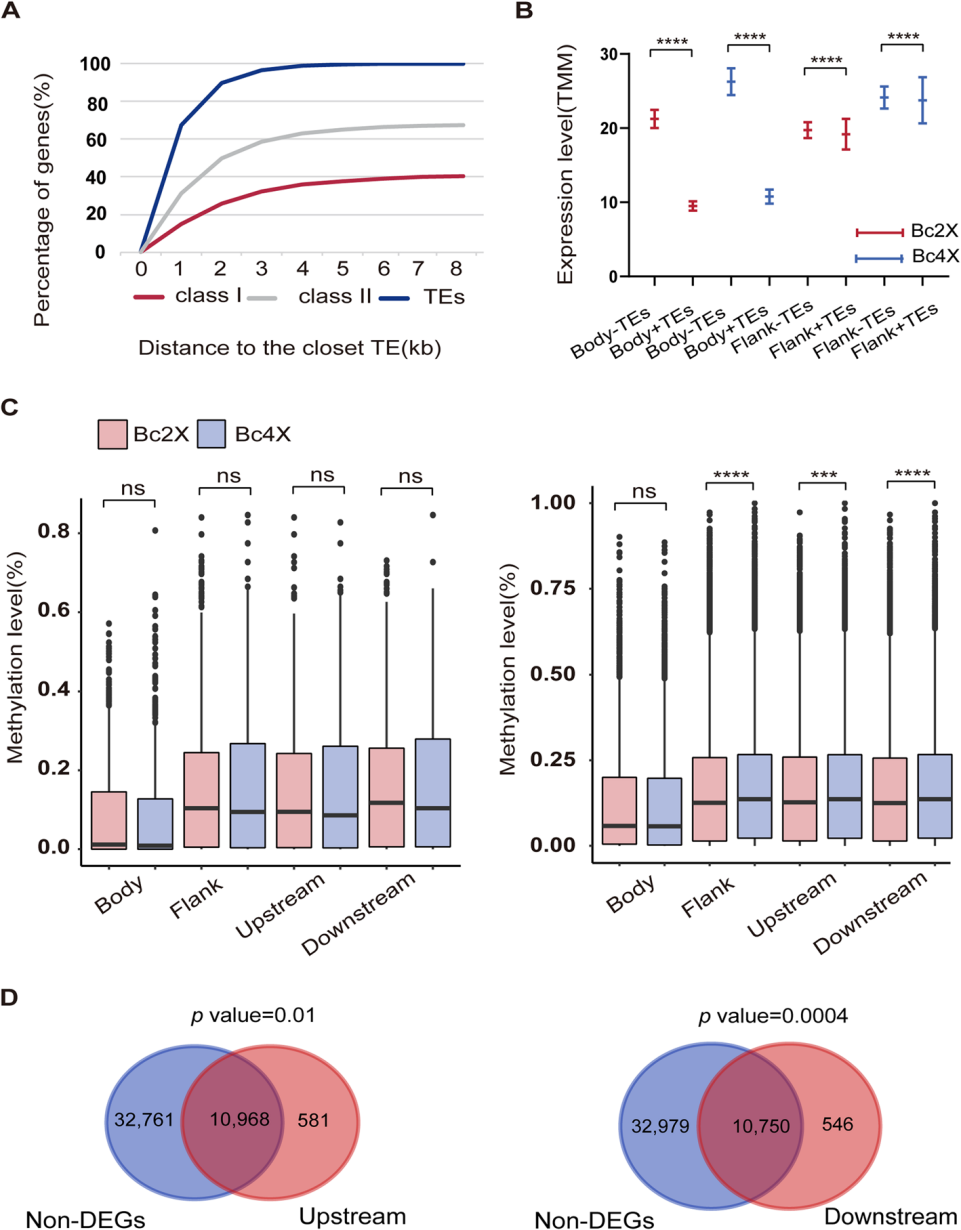
Relationship between non-DEGs and adjacent TE methylation changes. **A** The percentage of genes inserted by TEs in their bodies and flanking regions. **B** Transcription levels of genes with or without TEs. “Flank” indicates 1 kb flanking regions of the gene; “ + ” indicates TEs inserted in this region; “-” indicates TEs not inserted in this region. Statistical analysis was performed with two-tailed Wilcoxon tests. *P* value: “****” < 0.0001. **C** DNA methylation levels of TEs in gene bodies or 1 kb flanking regions (upstream or downstream) of DEGs (left panel) and non-DEGs (right panel). The *P* value was calculated via the two-sided Wilcoxon test. *P* value: “ns” means not significant; *P* value: “****” < 0.0001. The error bars indicate the SEMs. **D** Venn diagrams showing the overlaps between non-DEGs and genes with TEs in upstream or downstream regions. Significance was examined via the hypergeometric test

Given that the methylation level of TEs is much greater than that of genes, we explored the relationship between TE methylation and the transcription of TE-neighboring genes. First, differences in the methylation levels of TEs between Bc2X and Bc4X were detected for 12 major TE superfamilies belonging to two TE classes: class I (retrotransposons) and class II (DNA transposons). TEs in gene bodies are more likely to be class II TEs (including the *Hel, mul, hAT, Sto, En,* and *Har* superfamilies) than class I TEs (LTR retrotransposons, including *Gypsy, Copia* and nonLTR retrotransposons, including *LINEs* and *SINEs*) (Fig. 6A). In class I TEs, DNA methylation is significantly increased for *Copia* and *Gypsy.* Among class II TEs, DNA methylation was significantly increased in the *Sto* and *Har* TE superfamilies but decreased in the *hAT* and *En* superfamilies (Figure S9A-B). The average gene transcription level was positively correlated with the distance from the nearest TE, especially within the 1 kb flanking region (Figure S10A). To determine the effect of TEs on the transcription of TE-neighboring genes, we identified the gene bodies and 1 kb flanking regions inserted by TEs. In both Bc2X and Bc4X, the overall transcription level of genes with TEs was lower than that of genes without TEs (Fig. 6B). These results indicated that genome duplication altered the methylation levels of TEs and that TE insertion affected the transcription of TE-neighboring genes.

To further explore the effect of TE methylation on gene expression upon genome doubling, we compared the changes in TE methylation levels in 1 kb flanking regions and gene bodies of DEGs and non-DEGs. The results revealed that there was no significant change in the TE methylation of DEGs upon genome doubling, either in the body or flanking regions (Fig. 6C). However, a significant increase in TE methylation of non-DEGs occurred in the 1 kb flanking regions, both upstream and downstream (Fig. 6C). In addition, the genes with TEs inserted in 1 kb flanking regions (upstream and downstream) were significantly enriched with non-DEGs (Fig. 6D). Moreover, the average gene transcription level of DEGs/non-DEGs was positively correlated with the distance from the nearest TE within the 1 kb flanking region (Figure S10). Together, these results indicate that the methylation level of TEs flanking non-DEGs significantly increases upon genome doubling to maintain the transcriptional homeostasis of a large number of non-DEGs (94.55%).

## Discussion

Polyploidy occurs frequently in plants and has long been considered an important driver of evolution (Vandepoele et al. 2002). Even*Arabidopsis*, which has the smallest angiosperm genome, shows features of ancient WGD (Soltis et al. 2015). Polyploids exhibit evolutionary novelty as a result of the higher genetic diversity of polyploids than their diploid ancestors. This diversity at the genetic level manifests itself in phenotypic, physiological, and biochemical aspects. Polyploids are thought to exhibit adaptive plasticity, which has contributed to the evolution of plants, including major crop varieties such as wheat, potato and*brassica*(Ramírez-González et al. 2018). Allopolyploids have been studied more extensively than autotetraploids. In fact, the formation rate of autotetraploids is higher than that of allotetraploids. Compared with that of allopolyploids, the genetic background of autotetraploids is not affected by hybridization. Therefore, autotetraploids are more suitable for studying the regulatory mechanism of gene expression in polyploid species, which tend to exhibit larger organ phenotypes because their cells are normally larger. For example, autotetraploid*Arabidopsis*plants exhibit wider rosette leaves, a later flowering period, larger cell/plant sizes, and larger seeds (Li et al. 2012). Our study revealed that Bc4X leaves are rounder and shorter at 21 days after germination.

Plant genome duplication causes problems related to genomic instability. In allopolyploids, the incorporation of different parental genomes into a single nucleus may lead to the phenomenon of “genome shock” (McClintock and McClintock 1984). The response of plants to “genome shocks” may lead to rapid genome-wide changes, including changes in DNA methylation patterns (Bird et al. 2018). Autopolyploids must overcome the problem of meiotic adaptation caused by genome doubling, even if their nuclear environment changes are less complex than those of allopolyploids (Yant et al. 2013). Transcriptome analysis in this study revealed that genome duplication did not produce a large number of DEGs (approximately 5.45%) in pak choi, suggesting that there may be a counterbalancing mechanism to maintain transcriptional homeostasis in the duplicated genome.

Previous studies have reported that DNA methylation changes in autotetraploid plants. For example, CG and CHH methylation levels are significantly lower in the TEs of autotetraploid grape than in those of diploid grape (Xiang et al. 2023). Compared with diploid*Poncirus trifoliata*, autotetraploids undergo more extensive DNA demethylation under cold stress. There is no significant difference in the total DNA methylation level between diploid and autotetraploid watermelon, whereas autotriploid watermelon tends toward a lower level of DNA methylation (Wang et al. 2009). Our results revealed that the transcription levels of demethylases (*BcROS1*, *BcDME*) associated with DNA methylation are significantly reduced, and the CG, CHG and CHH methylation levels of Bc4X are all greater than those of Bc2X, which is similar to the pattern of DNA methylation variation in autotetraploid rice (Sun et al. 2022). In*Arabidopsis*, AtROS1 has been reported to antagonize the RdDM pathway, which is dependent on the multisubunit RNA polymerases Pol IV and Pol V. The transcription levels of Pol IV- and Pol V-related genes (*BcNRPD2*, *BcNRPD4*, *BcNRPE5*, *BcDRD1* and *BcDMS3*) are differentially regulated. In addition, our study revealed the differential expression of several genes that might be important for plant growth and development. For example, *BraA09g003290.3C* is homologous to the *Arabidopsis* gene *AT3G27690*, which encodes the light-harvesting chlorophyll a/b binding (LHC) protein LHCB2.4. After genome doubling, the downregulation of this gene may affect the growth and development of Bc4X; *BraA03g004720.3C* is homologous to *AT5G11530,* which encodes EMF1 for flower formation in *Arabidopsis*(Aubert et al. 2001), and the downregulation of this gene may be associated with the late-flowering phenotype of Bc4X (Zhang et al. 2020; Han et al. 2014). Previous studies revealed that the crosstalk between auxin and brassinosteroids can regulate leaf shape (Xiong et al. 2021), and the downregulation of*BcPIN4* and *BcBZR1*, which are homologous to *the Arabidopsis* genes encoding PIN4 and BZR1,respectively, may be associated with the more rounded leaf autotetraploid pak choi seedlings at 21 days after germination. In addition, previous reports have demonstrated that *linc1linc2*double-mutant plants present dwarf and curly leaf phenotypes (Dittmer et al. 2007). In this study, we detected a decreased transcription level of*BcLINC1 in Bc4X*, which may be involved in the slow growth rate of autotetraploid pak choi. In rice, studies have shown that methylation alterations caused by polyploidization and hybridization may affect gene expression (Rao et al. 2023). Our study revealed that DEGs overlap significantly with DMGs and that the transcription level of DEGs is negatively correlated with the methylation levels across these DMGs. There was no significant association between non-DEGs and DMGs. Previous findings in*Arabidopsis*suggested that the number, distance, and methylation status of TEs influence the expression of TE-neighboring genes (Hollister and Gaut 2009; Wang et al. 2013). In allopolyploid*Brassica napus*, the genomic asymmetric epigenetic modification of transposable elements is involved in the regulation of gene expression (Xiao et al. 2024). In rice autotetraploids, increased global TE methylation may inhibit gene expression (Zhang et al. 2015). We found that the non-DEGs accounted for a high proportion (94.55%) of the total genes in pak choi after genome doubling and revealed that the methylation of TEs flanking non-DEGs significantly increased, whereas this did not occur with the DEGs. This regulatory mechanism might be one of the reasons for the existence of many non-DEGs that escape from transcriptional imbalance. The increased TE methylation of flanking non-DEGs might suppress the potential transcriptional up-regulation of these non-DEGs upon genome doubling, thus overcoming the “genome shock” caused by genome duplication and maintaining the genome stability of autopolyploid pak choi.

In addition to DNA methylation, histone modifications have been reported to be involved in the regulation of gene expression after genome doubling in plants. For example, H3K4me3 and H3K27me3 are changed and involved in the regulation of transcription at specific genomic sites during *Arabidopsis*genome doubling (Zhang et al. 2019). Furthermore, the genomic dose-dependent alleles did not contain TEs and were associated with the commonly active markers H3K9ac and H3K4me3, whereas most of the dose-independent alleles were enriched for TEs, CHG methylation, and silencing markers such as H3K27me1 and H3K27me3 (Shi et al. 2015). It is therefore of interest to study comprehensively the transcriptional regulation during genome dosage changes by combining the roles of different epigenetic mechanisms.

## Methods

### Plant materials and growth conditions

The autotetraploid (Bc4X) pak choi was generated from Bc2X (*Brassica rapa* ssp. *Chinensis* cv. *Liuhe Aijiaohuang*) by colchicine and characterized by flow cytometry and mitotic chromosome spreading (Han et al. 2014). The sixth generation of Bc4X was used in this study. The plants were grown in a light incubator (12 h light/12 h dark photoperiod at 22 °C/18 °C, with 75% relative humidity) with pots containing a soil/sand mixture (3:1). Diploid and autotetraploid pak chois were grown under the same conditions, and the second leaves (from the top) were collected from 21-day-old seedlings at the “two-leaf” stage for transcriptome and DNA methylation sequencing.

### Identification of DNA methylation pathway-related genes

The sequences of DNA methylation pathway-related genes in *Arabidopsis*were first collected. On the basis of the BRAD database (Chen et al. 2022), BLASTP was used to compare the*Arabidopsis*protein sequences with those of pak choi, and the candidate proteins were screened according to the score value (Altschul et al. 1990). Next, the BLASTP search results were evaluated for statistical and biological significance to determine the potential orthologs related to DNA methylation. MEGA(v7.0) (Kumar et al. 2016) software was subsequently used to construct a phylogenetic tree via the amino acid sequences of the screened protein-coding genes via the maximum likelihood method to explore the evolutionary relationships between genes.

### RNA-seq and data analysis

Total RNA from leaves was isolated via TRIzol reagent (Invitrogen) following the manufacturer’s instructions. The purity of the extracted RNA was detected via a spectrophotometer (NanoDrop 2,000&8,000), and the concentration and integrity of the RNA were detected via a bioanalyzer (Agilent 2100, Agilent Technologies, Palo Alto, CA, USA). Three biological replicates of RNA-seq libraries were constructed for each group. Sequencing was performed via an Illumina platform (NovaSeq) at the Igenecode Company (Beijing, China), and paired reads with an average length of 150 bp were generated. Clean data were obtained after filtering reads with >  = 5 N bases, a phred quality value < 20, and lengths < 75 bp and removing adapter sequences via fastp (v0.22.0) (Ondov et al. 2016). After data quality control, the clean reads were mapped to the*B. rapa*genome (v3.0) via HISAT2 (v2.0.4) (Kim et al. 2019) with parameters: hisat2 -p 10 -X−1 −2-S –dta. The mapped reads were converted into transcripts via featureCounts (v2.0.3) (Liao et al. 2014)with parameters: featureCounts -p -T 6 -t exon -g gene, and the read count number was calculated. Deseq2 package (v1.36.0) (Love et al. 2014) was used for differential gene detection, with a fold change >  = 2.00 and adjusted*p*value <  = 0.01. Gene function annotation was performed via eggNOG-Mapper (v2.0) (Cantalapiedra et al. 2021) athttp://eggnog-mapper.embl.de/.The eggNOG-mapper annotation results were used in the AnnotationForge package (v1.44.0) to construct the Orgdb package, which was used for GO annotation. KEGG and GO annotation analyses were performed by using clusterProfiler package (v4.0) (Wu 2021). The heatmap was generated using the pheatmap package (v1.0.12), and the data in each column were normalized by Z-score.

### WGBS and analysis

The plant samples used for WGBS were the same as those used for RNA-seq, as described above. The leaf tissues were used for genomic DNA extraction via a CTAB-based protocol with two biological replicates. Quantification was performed with an Agilent 2100 spectrophotometer. Bisulfite treatment (EZ DNA Methylation-Gold Kit, Zymo, D05005), library construction (TruSeq DNA Methylation Kit, Illumina, EGMK91396), and sequencing (iIIumina Novaseq6,000) were performed at Oebiotech Company (Shanghai, China). Clean data were obtained after filtering reads with >  = 5 N bases, phred quality values < 20, and lengths < 75 bp and removing adapter sequences with the fastp package (v0.22.0) (Ondov et al. 2016). After quality control, Bismark (v2.5.1) (Krueger et al. 2011) was used to map the clean data to the*B. rapa* genome (v3.0), the parameters were Bismark –bowtie2 –parallel 40 –genome −1 −2. After mapping, cytosine methylation sites were determined, and methylation levels were calculated via Batmeth2 (Zhou et al. 2019). To ensure the accuracy of methylation site detection, sites with >  = 10 covered reads were taken as the truly detected C sites with the following parameters: Batmeth2 calmeth -Q 20 –remove_dup –coverage 10 -nC 1 –Regions 1,000 –genome –binput –methratio. Each context of methylation was considered independently: CG, CHG or CHH. To calculate methylation levels along chromosomes, the genome was first slide windowized with the BEDtools (v2.30.0) (Quinlan et al. 2010) tool with the following parameters: bedtools makewindows –window 500,000 –step 100,000. After that, the methylation levels of the windows were calculated via the BatMeth2 MethyGff tool, whose input file includes the results of the site methylation level described above, and the parameters were BatMeth2 methyGff –coverage 10 -nC 1 –genome -gff –body –promoter –GENE –distance 1,000 –methratio.

### 5-Aza treatment and Chop-PCR

After germination, Bc2X and Bc4X were transferred to 1/2 MS containing the DNA methylation inhibitor 5-Aza (100 µM) in a light incubator (12 h light/12 h dark photoperiod at 22 °C/18 °C, with 75% relative humidity). Leaves were randomly collected from each treatment group, avoiding veins, and samples were snap frozen in liquid nitrogen and then stored at −80 °C for the following experiments. One microgram of genomic DNA was digested with the methylation-sensitive enzyme *Hpa*II in a 20 μl reaction mixture. After digestion, PCR was performed via the use of 1 μl of the digested DNA as a template in a 10 μl reaction mixture and the use of primers for 1 kb differential CG methylated regions (Dasgupta et al. 2019). The primers used are listed in Table S6.

### Analysis of DMRs and DMGs

DMRs were defined as the specific regions on the genome where methylation levels varied significantly between Bc2X and Bc4X, and DMGs were defined as genes whose gene bodies, promoters and downstream regions overlapped with the DMRs. To obtain reliable DMRs, replicates from each sample were merged. DMRs were identified with the sliding-window approach. Briefly, methylation levels of different cytosine sites within 1000 bp tiling windows were integrated with > 10 × coverage using methylKit (v1.10.0) (Akalin et al. 2012). Logistic regression was used to detect significant methylation changes, with a*P* value cutoff of 0.05. Methylkit will correct *P*values to Q values via the sliding linear model (SLIM) approach to correct the multiple hypothesis testing problem. The three context sequence (CG, CHG or CHH) DMRs were calculated separately and filtered with Q values < 0.05 and differential methylation levels (Meth_Diff) >  = 10%. GO annotation analyses of the genes that overlapped between the DMGs and DEGs were performed via ClusterProfiler (v4.0) (Wu 2021).

### TE analysis

TE annotations were obtained from BRAD, and the distances between genes and TEs were calculated via ChIPseeker package (Yu et al. 2015). The methylation levels of TEs were calculated using BatMeth2 (Zhou et al. 2019), with parameters: BatMeth2 methyGff –coverage 10 -nC 1 –genome -gff –body –promoter –GENE –distance 1000 –methratio.

## Supplementary Information

Additional file 1. Figure S1. Data quality control for the Bc2X and Bc4X RNA-seq experiments. (A) Sample hierarchical cluster analysis of Bc2X and Bc4X. (B) Heatmap showing the clustered gene transcription profiles of the biological replicates in A. (C) PCA plot of gene transcription in the RNA-seq data of Bc2X and Bc4X. (D) qRT‒PCR results for several genes. The normalized gene transcription levels were arbitrarily set to 1 for Bc2X. Each bar shows the mean ± SEM of triplicate assays. “**” indicates a statistically significant difference relative to the value at Bc2X for each gene at a *p* value < 0.01. Statistical analysis was performed with two-tailed Student’s *t* tests. (E) Global transcription levels of genes between Bc2X and Bc4X.

Additional file 2. Figure S2. Transcription levels of genes between Bc2X and BC4X in all chromosomes (A01-A10). The heatmap shows the comparisons of gene transcription levels between Bc2X and Bc4X in the chromosomes indicated. Each horizontal line in the individual chromosome represents a gene with the transcription level normalized to Z-score. For each chromosome, the left panel represents Bc2X, and the right panel represents Bc4X.

Additional file 3. Figure S3. Transcription levels of genes involved in the RdDM pathway. (A) Heatmaps showing the relative transcription levels of genes related to the RdDM pathway in Bc2X and Bc4X, with three biological replicates. The upper panel shows the Pol IV- and Pol V-related genes, and the lower panel shows the RdDM pathway-related genes. (B) Transcription levels of sixteen RdDM pathway-related genes. Statistical analysis was performed with two-tailed Student’s *t* tests. *P* value: “**” < 0.01, “*” < 0.05.

Additional file 4. Figure S4. Transcription levels of genes involved in DNA methylation and demethylation pathways. (A) Phylogenetic analysis of DNA methyltransferase (upper panel) and demethylase genes (lower panel) in the *B. rapa* genome. (B) Heatmaps showing the relative transcription levels of DNA methyltransferase (upper panel) and demethylase genes (lower panel) in Bc2X and Bc4X with three biological replicates. (C) Transcription levels of four DNA methylation-related enzymes (*BcCMT3*, *BcDRM2-2*, *BcROS1* and *BcDME*). Statistical analysis was performed with two-tailed Student’s *t* tests. *P* value: “*” < 0.05. The trimmed mean of M values (TMM) is a way to standardize the relative transcription level.

Additional file 5. Figure S5. Characterization of DNA methylation levels in the Bc4X genome. The plots showing DNA methylation in three sequence contexts (CG, CHG, CHH) of Bc4X and the densities of genes and TEs across chromosomes.

Additional file 6. Figure S6. Genome-wide densities of different subcontexts. (A) Boxplots showing the densities of each subcontext across chromosomes per 500 kb bin. (B) Densities of CG, CHG and CHH subcontexts across chromosomes per 500 kb bin.

Additional file 7. Figure S7. Relationship between the methylation and transcription of genes. (A) Venn diagrams showing the overlaps between the downregulated genes and DMGs with altered DNA methylation in gene bodies or 1 kb flanking regions. Significance was examined via the hypergeometric test. (B) Venn diagrams showing the overlaps between the non-DEGs and DMGs (hyper/hypoDMGs). Significance was examined via the hypergeometric test. (C) Scatter plots showing the correlations between CG, CHG, and CHH methylation and the transcription of significantly enriched genes between DEGs and DMGs. Statistical analysis was performed with two-tailed Student’s *t* tests. (D) GO analysis of genes significantly enriched between DEGs and DMGs in the CG context.

Additional file 8. Figure S8. Relationship between differential DNA methylation and gene transcription upon genome doubling. Chop-PCR was used to detect the DNA methylation status in 1 kb regions flanking genes, including *BcPIN4* (*BraA02g034640.3C*), *BcLINC1* (*BraA07g032580.3C*), and *BcBZR1* (*BraA07g027940.3C*), after 5-Aza treatment. Bc2X and Bc4X plants with ( +) or without (-) 5-Aza treatment were used as samples for Chop-PCR testing. The gel plots show the CG methylation status of different samples digested with ( +) or without (-) the specific methylation-sensitive enzyme *Hpa*II. The undigested region of Bc2X was used as a control. The qRT‒PCR results shown in the bar charts represent the relative transcription levels of the indicated genes before and after 5-Aza treatment of Bc2X and Bc4X. Statistical analysis was performed with two-tailed Student’s *t* tests. *P* value: “****” < 0.0001, “*” < 0.01. The error bars indicate the means ± SEMs.

Additional file 9. Figure S9. The genome-wide DNA methylation levels of major class I and class II TEs. (A) Genome methylation levels of class I TEs. Statistical analysis was performed with two-tailed Wilcoxon tests. *P* value: “****” < 0.0001, “**” < 0.01, “ns” not significant. (B) Genome methylation levels of class II TEs. Statistical analysis was performed with two-tailed Wilcoxon tests. *P* value: “**” < 0.01. *P* value: “****” < 0.0001, “ns” not significant.

Additional file 10. Figure S10. Transcription levels of genes related to the distance to the closest TE in Bc2X and Bc4X. (A) Transcription levels of DEGs related to the distance to the closest TE in Bc2X and Bc4X. “0” indicates genes overlapping with TEs in gene bodies. The error bars indicate the SEMs [Pearson’s r (Bc2X) = 0.053, *P* value = 0.003; r (Bc4X) = 0.017, *P* value = 0.004]. (B) Transcription levels of non-DEGs related to the distance to the closest TE in Bc2X and Bc4X. “0” indicates genes overlapping with TEs in gene bodies. The error bars indicate SEMs [Pearson’s r (Bc2X) = 0.016, *P* value = 0.005; r (Bc4X) = 0.015, *P* value = 0.01].

Additional file 11. Table S1. Statistics of the read quantity and quality of the filtered transcriptome sequencing data.

Additional file 12. Table S2. The transcription levels of all genes in Bc2X and Bc4X. Bc2X-1, Bc2X-2, and Bc2X-3 represent different biological replicates of Bc2X. Bc4X-1, Bc4X-2, and Bc4X-3 represent different biological replicates of Bc4X.

Additional file 13. Table S3. Transcription levels of polymerase-specific subunits in Bc2X and Bc4X and their homologs in *Arabidopsis*. Columns 5 to 10 present the transcription levels of Bc2X and Bc4X in three biological replicates. Statistical analysis was performed with two-tailed Student’s *t* tests. *P* value: “**” < 0.01, “ns” is not significant.

Additional file 14. Table S4. Transcription levels of DNA methylation-related genes in Bc2X and Bc4X and their homologs in *Arabidopsis*. Columns 5 to 10 present the transcription levels of Bc2X and Bc4X in three biological replicates. Statistical analysis was performed with two-tailed Student’s* t* tests. *P* value: “**” < 0.01, “ns” is not significant.

Additional file 15. Table S5. Statistics of read quantity and quality of filtered DNA methylation sequencing data.

Additional file 16. Table S6. Primers used in this study.

## Data Availability

A transcriptome assay was conducted by Boyun Huakang Gene Technology Co., Ltd. (Beijing, China). WGBS was obtained from Oebiotech Co., Ltd. (Shanghai, China). The raw RNA-seq and WGBS data used in this study have been uploaded to NCBI. Moreover, all other data are available from the corresponding author upon reasonable request.

## Abbreviations

Bc2X: Diploid Pak Choi
Bc4X: Autotetraploid Pak Choi
CMT3: Chromomethylase 3
DCL3: Dicer-Like Protein 3
DDM1: Decreased DNA Methylation 1
DMS3: Defective In Meristem Silencing 3
DRD1: Defective In RNA-directed DNA Methylation 1
DRM2: Domains Rearranged Methylase 2
DEG: Differentially Expressed Gene
DMC: Differentially Methylated Cytosine
DMR: Differentially Methylated Region
DMG: Differentially Methylated Region-Related Gene
GO: Gene Ontology
HDA6: Histone Deacetylase 6
hyperDMR: Differentially Hypermethylated Region
hypoDMR: Differentially Hypomethylated Region
hyperDMG: Differentially Hypermethylated Gene
hypoDMG: Differentially Hypomethylated Gene
hyperDMC: Hypermethylated DMC
hypoDMC: Hypomethylated-DMC
IDN2: Involved In *De Novo *2
IGV: Integrative Genomics Viewer
JMJ14: Jumonji 14
KEGG: Kyoto Encyclopedia of Genes and Genomes
LDL1: Lysine-Specific Demethylase 1-Like 1
MET1: Methyltransferase 1
MOM1: Morpheus Molecule 1
NCBI: National Center for Biotechnology Information
NRPD2: DNA-Directed Pol IV Subunit 2
NRPE5: DNA-Directed Pol V Subunit 5
qRT‒PCR: Quantitative Real-Time Polymerase Chain Reaction
RdDM: RNA-directed DNA methylation
RNA-seq: RNA sequencing
SUVR2: Dicer-Like Protein 3
TE: Transposable Element
TTS: Transcription Termination Site
TSS: Transcription Start Site
TAD: Topologically Associated Domain
WGD: Whole-Genome Duplication
WGBS: Whole-Genome Bisulfite Sequencing
