# Supplementary figures and images for: Regulation of transcriptional homeostasis by DNA methylation upon genome duplication in pak choi
Source: Mol Hortic. 2025 Apr 5;5:22. doi: 10.1186/s43897-025-00145-3 (PMC11971760; doi:10.1186/s43897-025-00145-3)

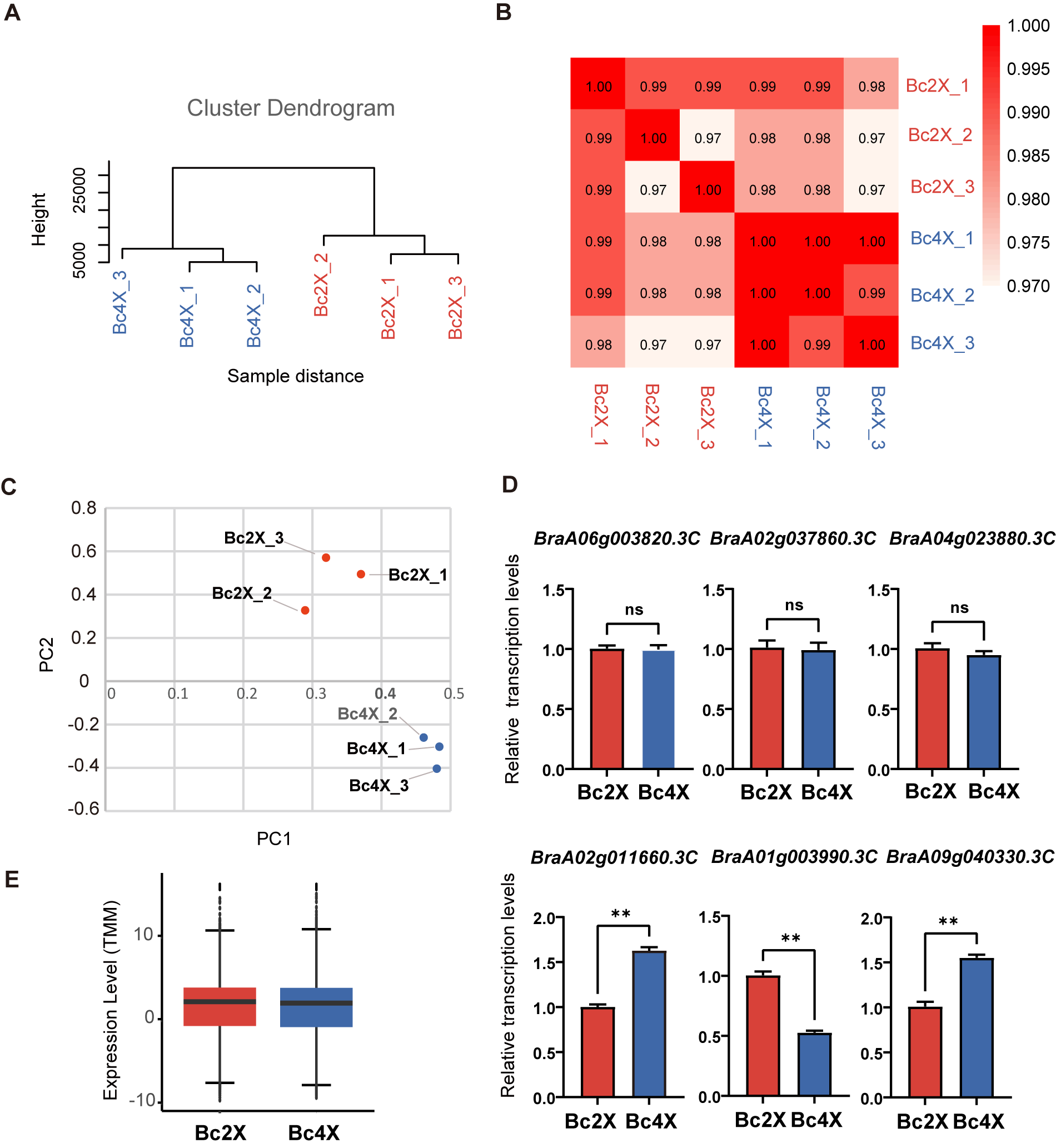

Supplement: Supplementary file 1 — Additional file 1. Figure S1. Data quality control for the Bc2X and Bc4X RNA-seq experiments. (A) Sample hierarchical cluster analysis of Bc2X and Bc4X. (B) Heatmap showing the clustered gene transcription profiles of the biological replicates in A. (C) PCA plot of gene transcription in the RNA-seq data of Bc2X and Bc4X. (D) qRT‒PCR results for several genes. The normalized gene transcription levels were arbitrarily set to 1 for Bc2X. Each bar shows the mean ± SEM of triplicate assays. “**” indicates a statistically significant difference relative to the value at Bc2X for each gene at a p value < 0.01. Statistical analysis was performed with two-tailed Student’s t tests. (E) Global transcription levels of genes between Bc2X and Bc4X. [file 43897_2025_145_MOESM1_ESM.tif]

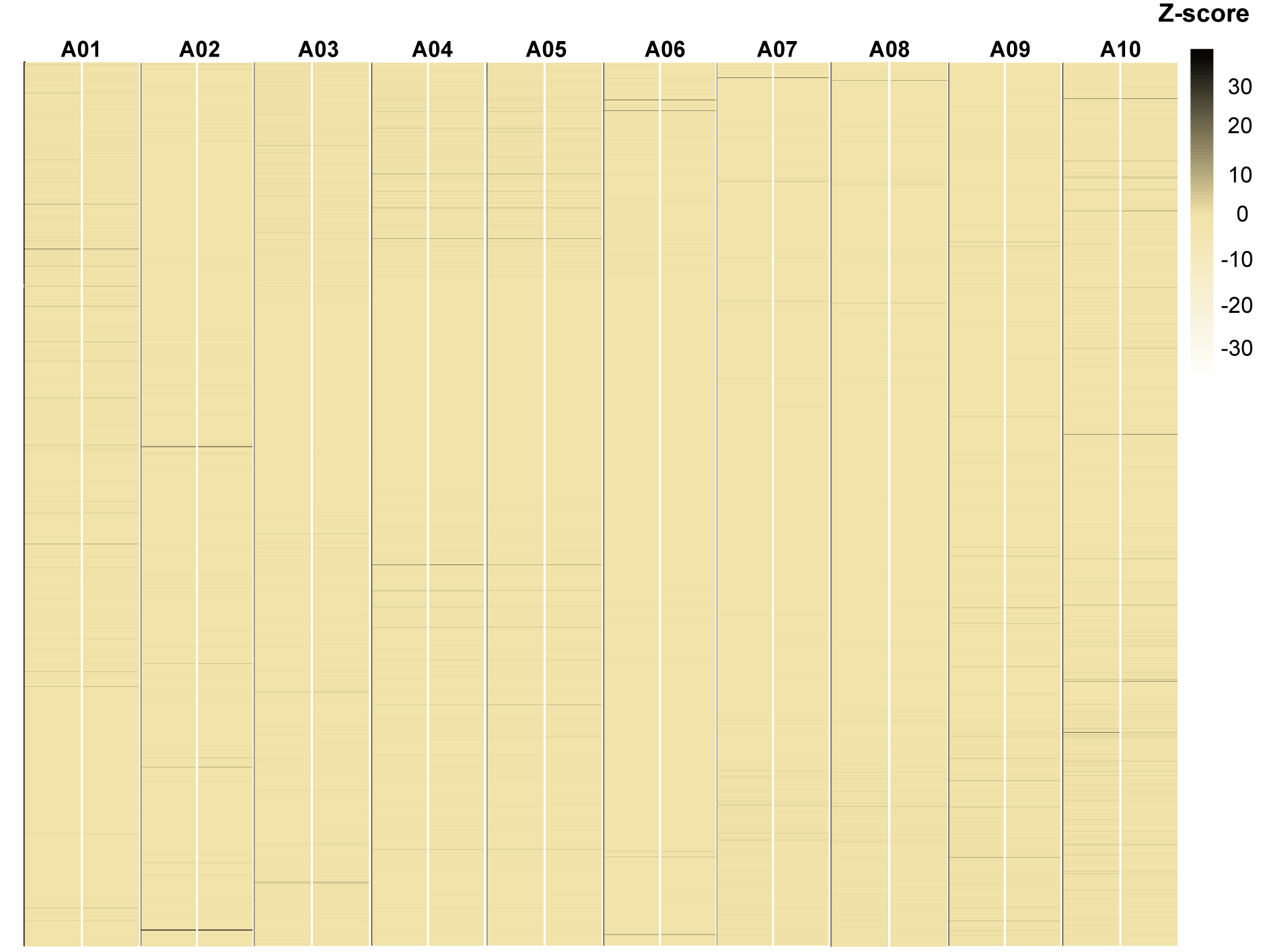

Supplement: Supplementary file 2 — Additional file 2. Figure S2. Transcription levels of genes between Bc2X and BC4X in all chromosomes (A01-A10). The heatmap shows the comparisons of gene transcription levels between Bc2X and Bc4X in the chromosomes indicated. Each horizontal line in the individual chromosome represents a gene with the transcription level normalized to Z-score. For each chromosome, the left panel represents Bc2X, and the right panel represents Bc4X. [file 43897_2025_145_MOESM2_ESM.tif]

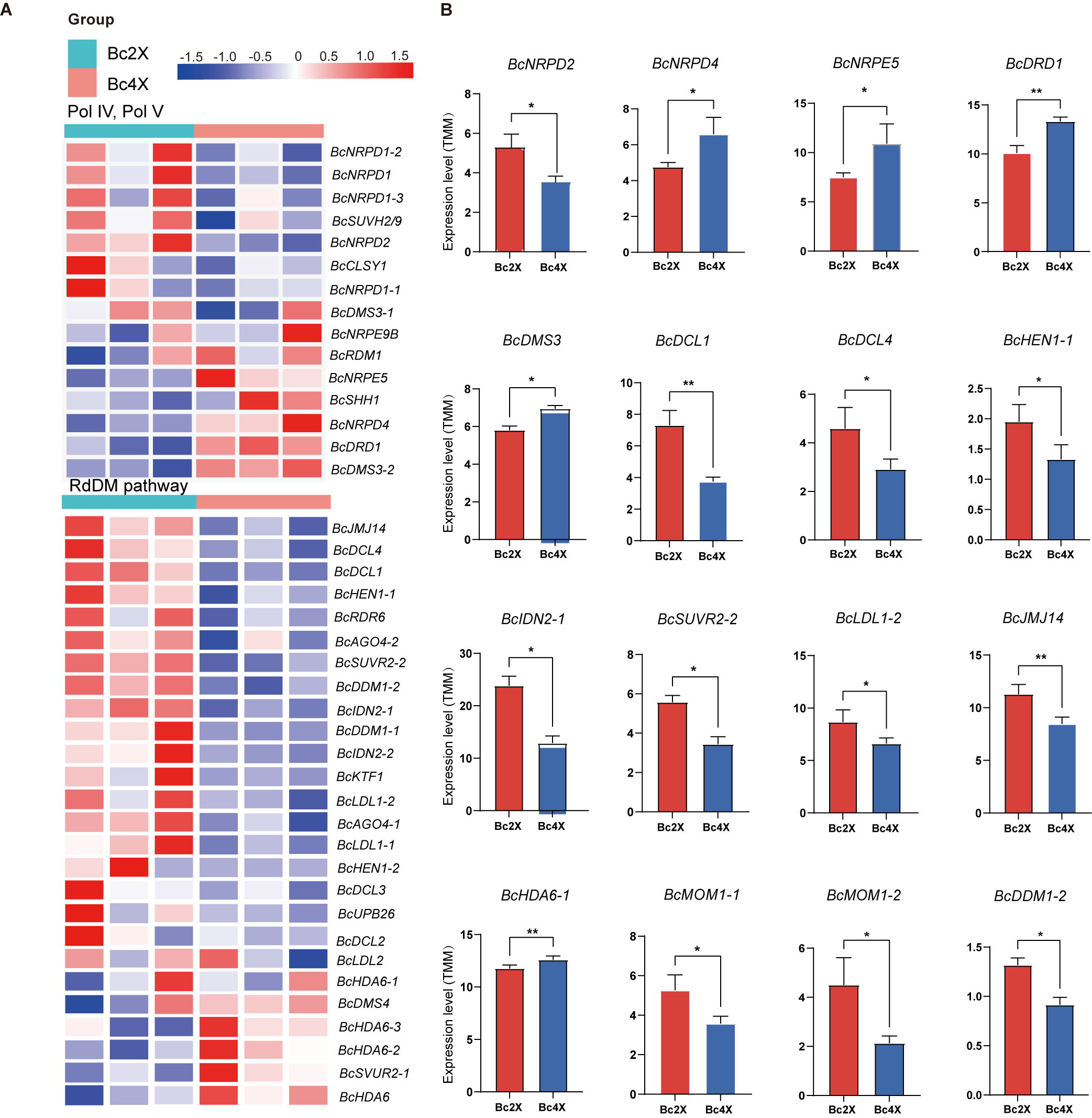

Supplement: Supplementary file 3 — Additional file 3. Figure S3. Transcription levels of genes involved in the RdDM pathway. (A) Heatmaps showing the relative transcription levels of genes related to the RdDM pathway in Bc2X and Bc4X, with three biological replicates. The upper panel shows the Pol IV- and Pol V-related genes, and the lower panel shows the RdDM pathway-related genes. (B) Transcription levels of sixteen RdDM pathway-related genes. Statistical analysis was performed with two-tailed Student’s t tests. P value: “**” < 0.01, “*” < 0.05. [file 43897_2025_145_MOESM3_ESM.tif]

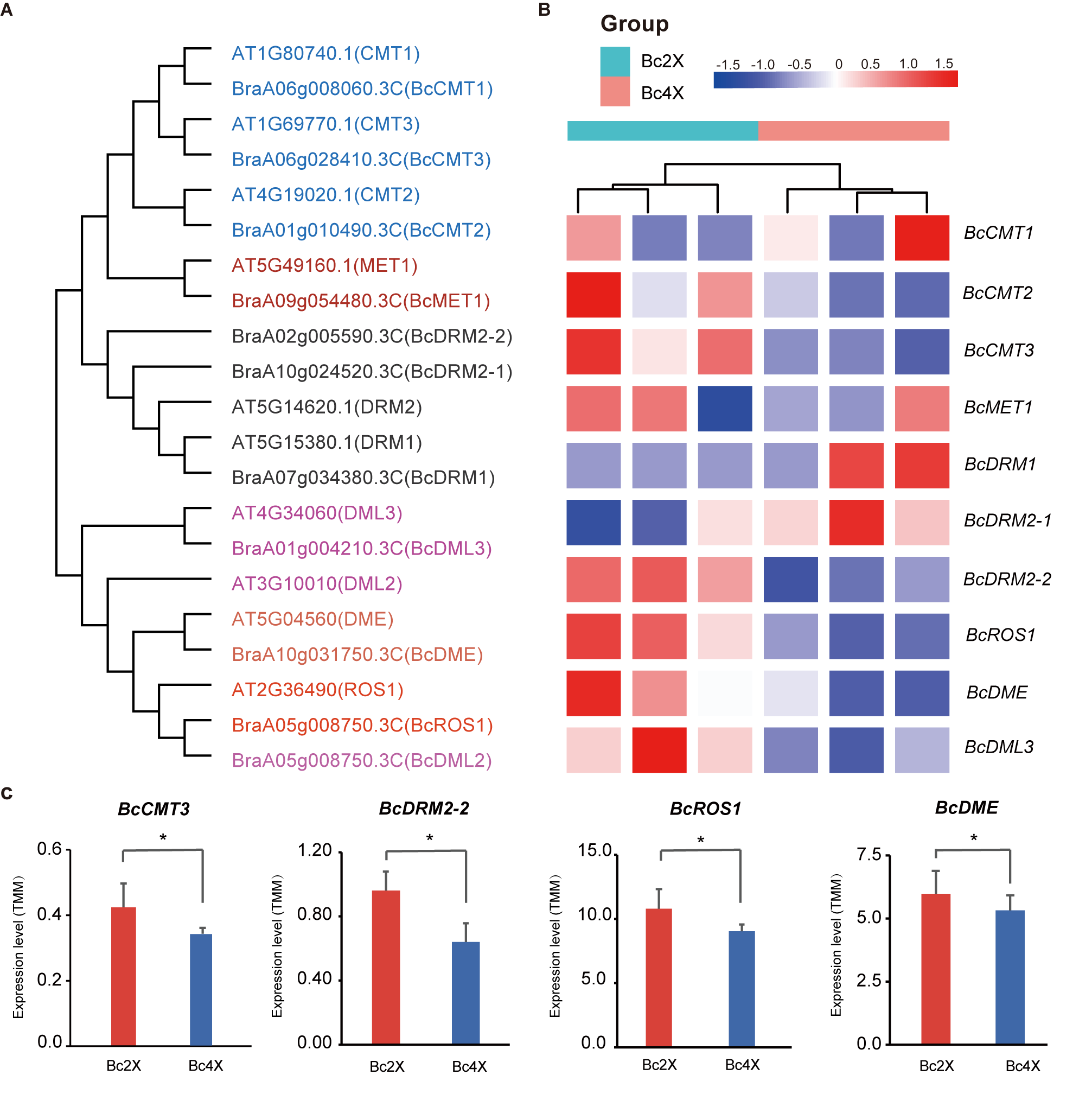

Supplement: Supplementary file 4 — Additional file 4. Figure S4. Transcription levels of genes involved in DNA methylation and demethylation pathways. (A) Phylogenetic analysis of DNA methyltransferase (upper panel) and demethylase genes (lower panel) in the B. rapa genome. (B) Heatmaps showing the relative transcription levels of DNA methyltransferase (upper panel) and demethylase genes (lower panel) in Bc2X and Bc4X with three biological replicates. (C) Transcription levels of four DNA methylation-related enzymes (BcCMT3, BcDRM2-2, BcROS1 and BcDME). Statistical analysis was performed with two-tailed Student’s t tests. P value: “*” < 0.05. The trimmed mean of M values (TMM) is a way to standardize the relative transcription level. [file 43897_2025_145_MOESM4_ESM.tif]

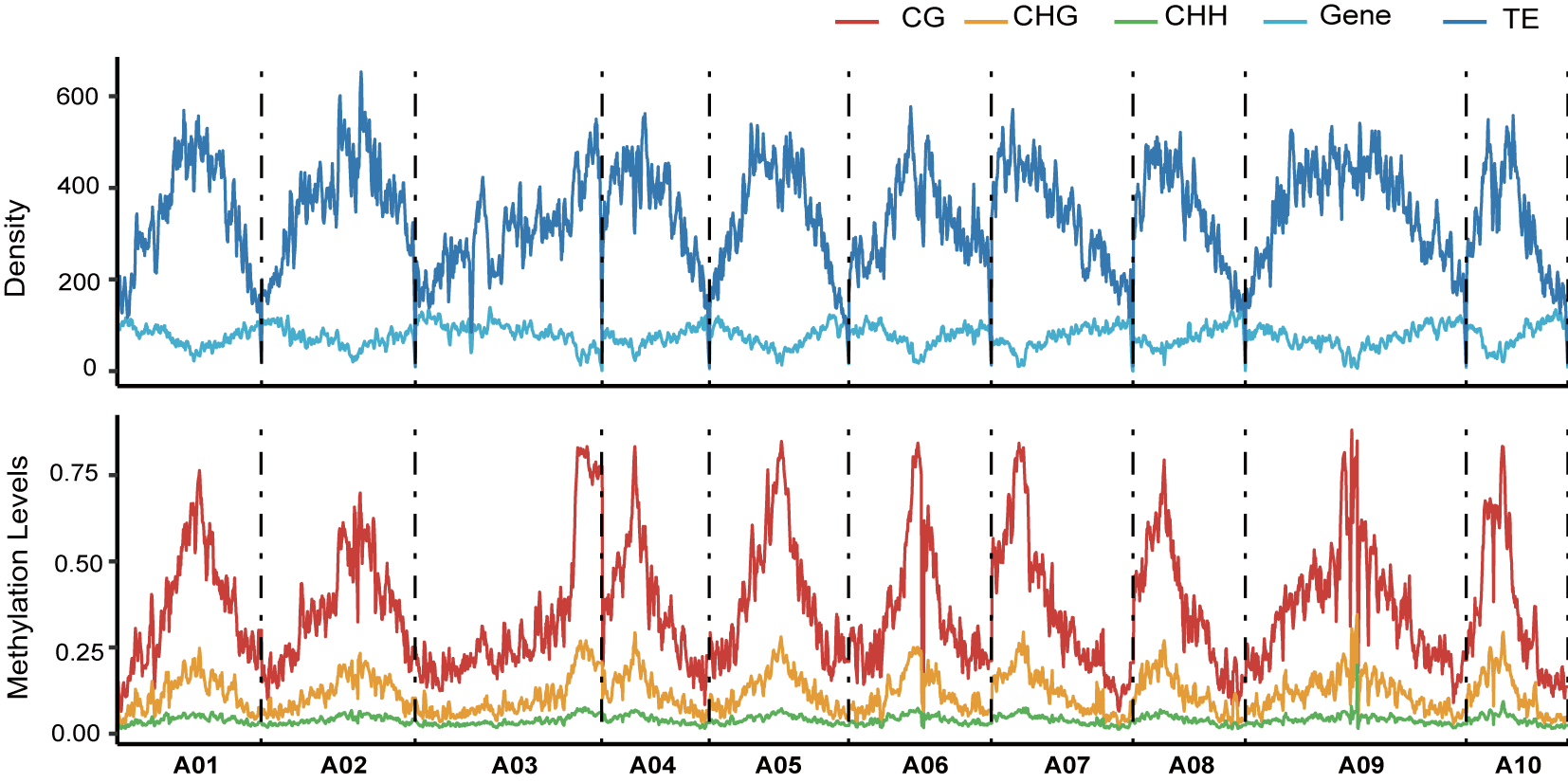

Supplement: Supplementary file 5 — Additional file 5. Figure S5. Characterization of DNA methylation levels in the Bc4X genome. The plots showing DNA methylation in three sequence contexts (CG, CHG, CHH) of Bc4X and the densities of genes and TEs across chromosomes. [file 43897_2025_145_MOESM5_ESM.tif]

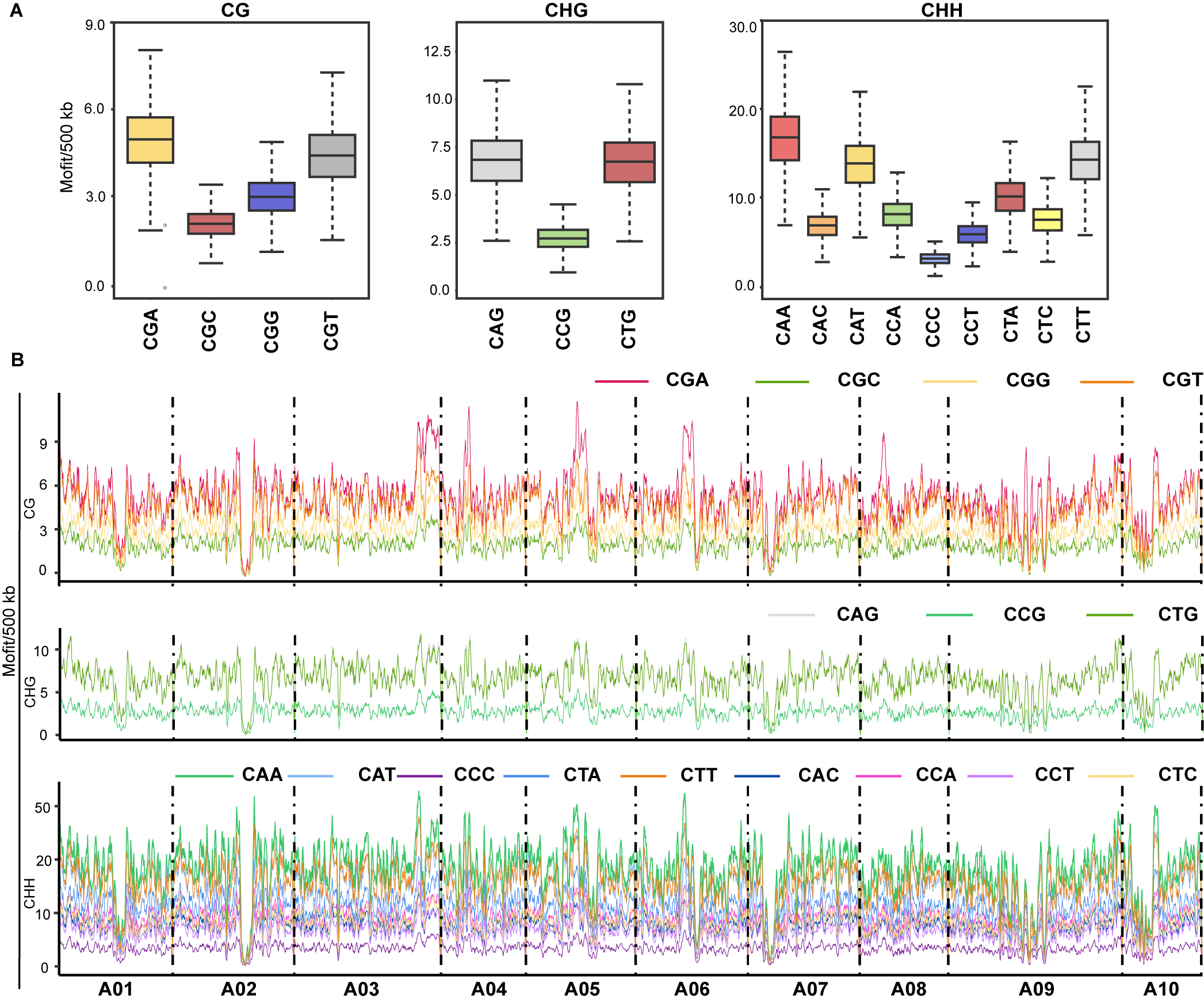

Supplement: Supplementary file 6 — Additional file 6. Figure S6. Genome-wide densities of different subcontexts. (A) Boxplots showing the densities of each subcontext across chromosomes per 500 kb bin. (B) Densities of CG, CHG and CHH subcontexts across chromosomes per 500 kb bin. [file 43897_2025_145_MOESM6_ESM.tif]

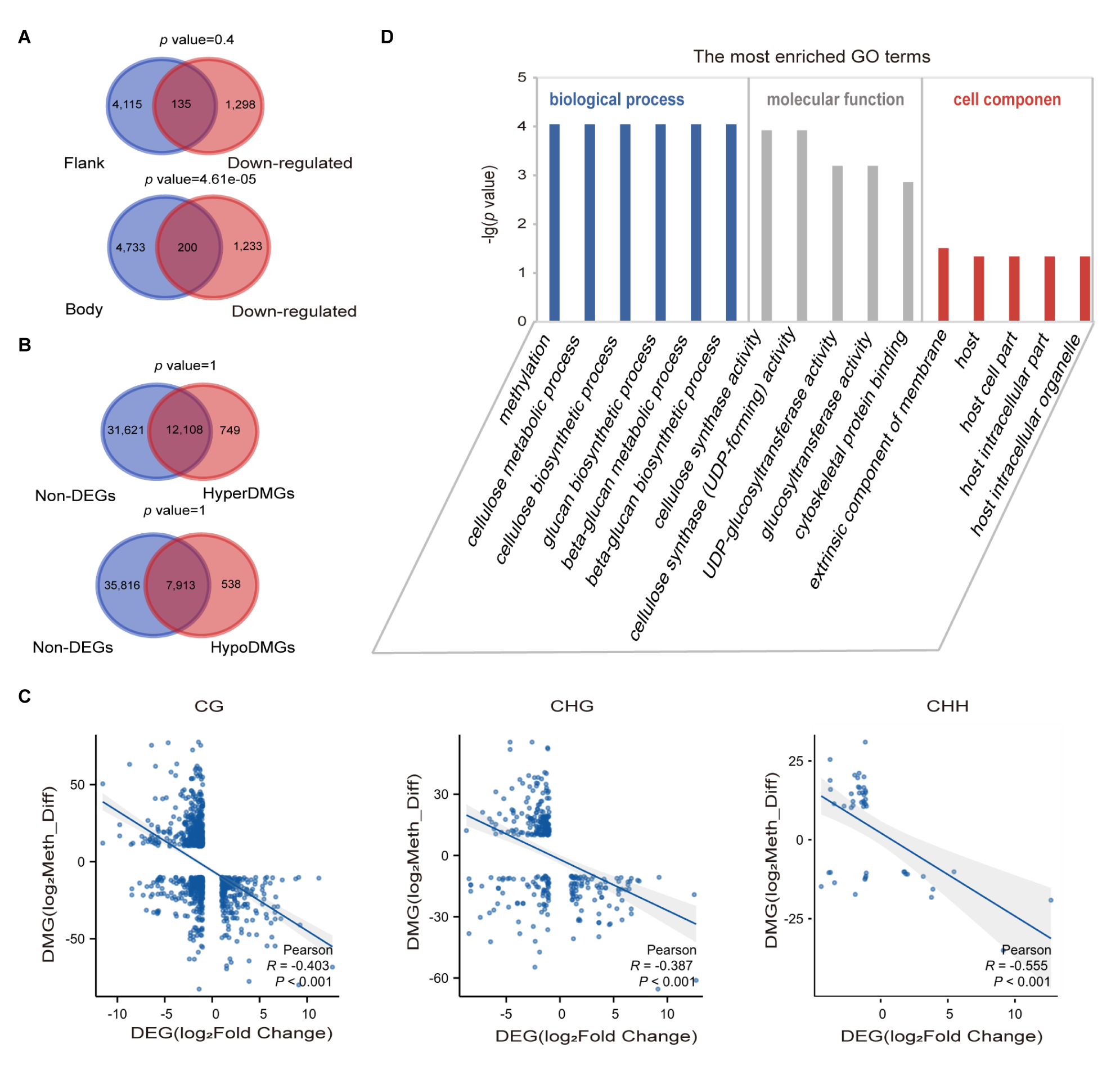

Supplement: Supplementary file 7 — Additional file 7. Figure S7. Relationship between the methylation and transcription of genes. (A) Venn diagrams showing the overlaps between the downregulated genes and DMGs with altered DNA methylation in gene bodies or 1 kb flanking regions. Significance was examined via the hypergeometric test. (B) Venn diagrams showing the overlaps between the non-DEGs and DMGs (hyper/hypoDMGs). Significance was examined via the hypergeometric test. (C) Scatter plots showing the correlations between CG, CHG, and CHH methylation and the transcription of significantly enriched genes between DEGs and DMGs. Statistical analysis was performed with two-tailed Student’s t tests. (D) GO analysis of genes significantly enriched between DEGs and DMGs in the CG context. [file 43897_2025_145_MOESM7_ESM.tif]

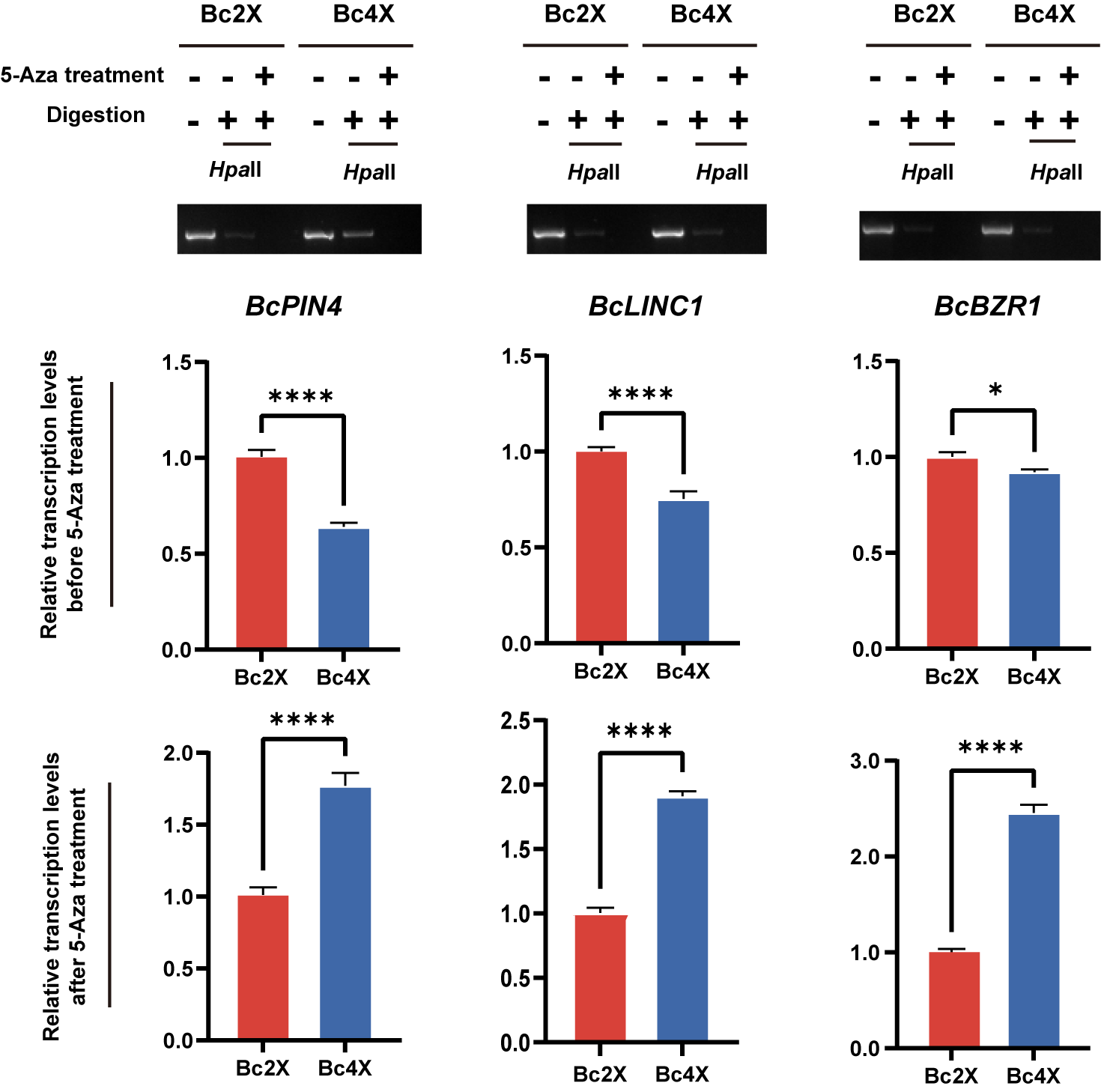

Supplement: Supplementary file 8 — Additional file 8. Figure S8. Relationship between differential DNA methylation and gene transcription upon genome doubling. Chop-PCR was used to detect the DNA methylation status in 1 kb regions flanking genes, including BcPIN4 (BraA02g034640.3C), BcLINC1 (BraA07g032580.3C), and BcBZR1 (BraA07g027940.3C), after 5-Aza treatment. Bc2X and Bc4X plants with ( +) or without (-) 5-Aza treatment were used as samples for Chop-PCR testing. The gel plots show the CG methylation status of different samples digested with ( +) or without (-) the specific methylation-sensitive enzyme HpaII. The undigested region of Bc2X was used as a control. The qRT‒PCR results shown in the bar charts represent the relative transcription levels of the indicated genes before and after 5-Aza treatment of Bc2X and Bc4X. Statistical analysis was performed with two-tailed Student’s t tests. P value: “****” < 0.0001, “*” < 0.01. The error bars indicate the means ± SEMs. [file 43897_2025_145_MOESM8_ESM.tif]

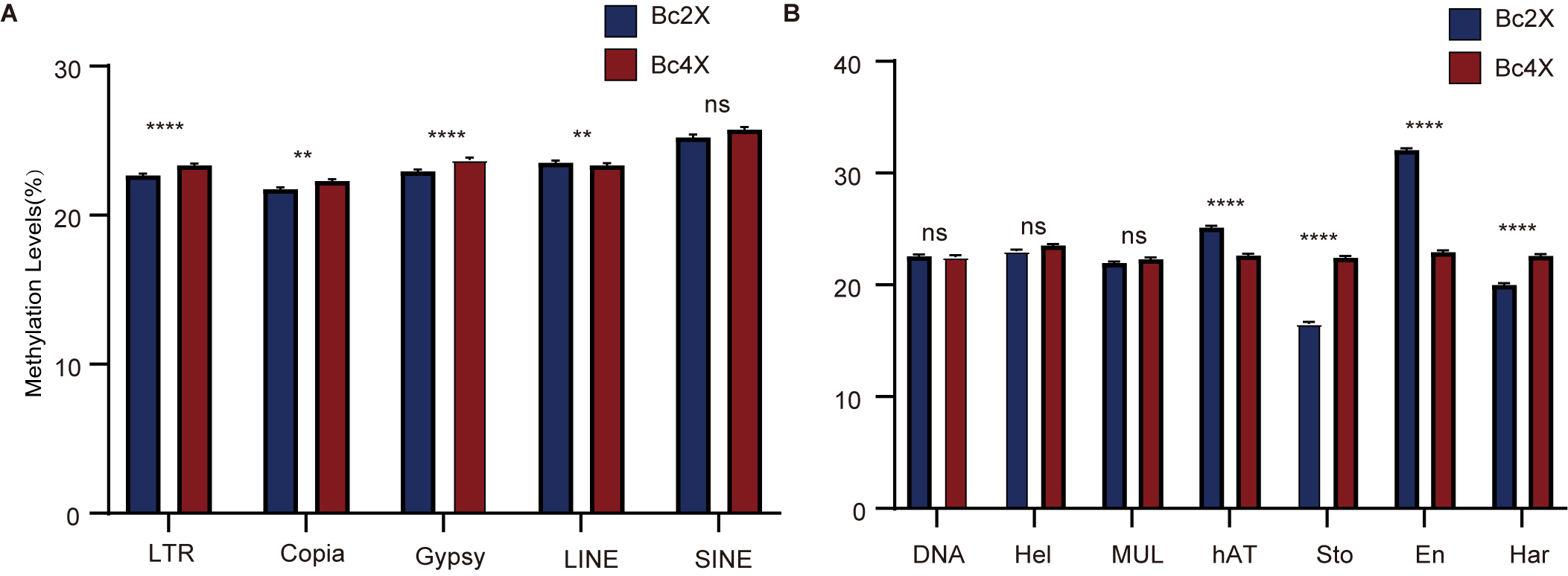

Supplement: Supplementary file 9 — Additional file 9. Figure S9. The genome-wide DNA methylation levels of major class I and class II TEs. (A) Genome methylation levels of class I TEs. Statistical analysis was performed with two-tailed Wilcoxon tests. P value: “****” < 0.0001, “**” < 0.01, “ns” not significant. (B) Genome methylation levels of class II TEs. Statistical analysis was performed with two-tailed Wilcoxon tests. P value: “**” < 0.01. P value: “****” < 0.0001, “ns” not significant. [file 43897_2025_145_MOESM9_ESM.tif]

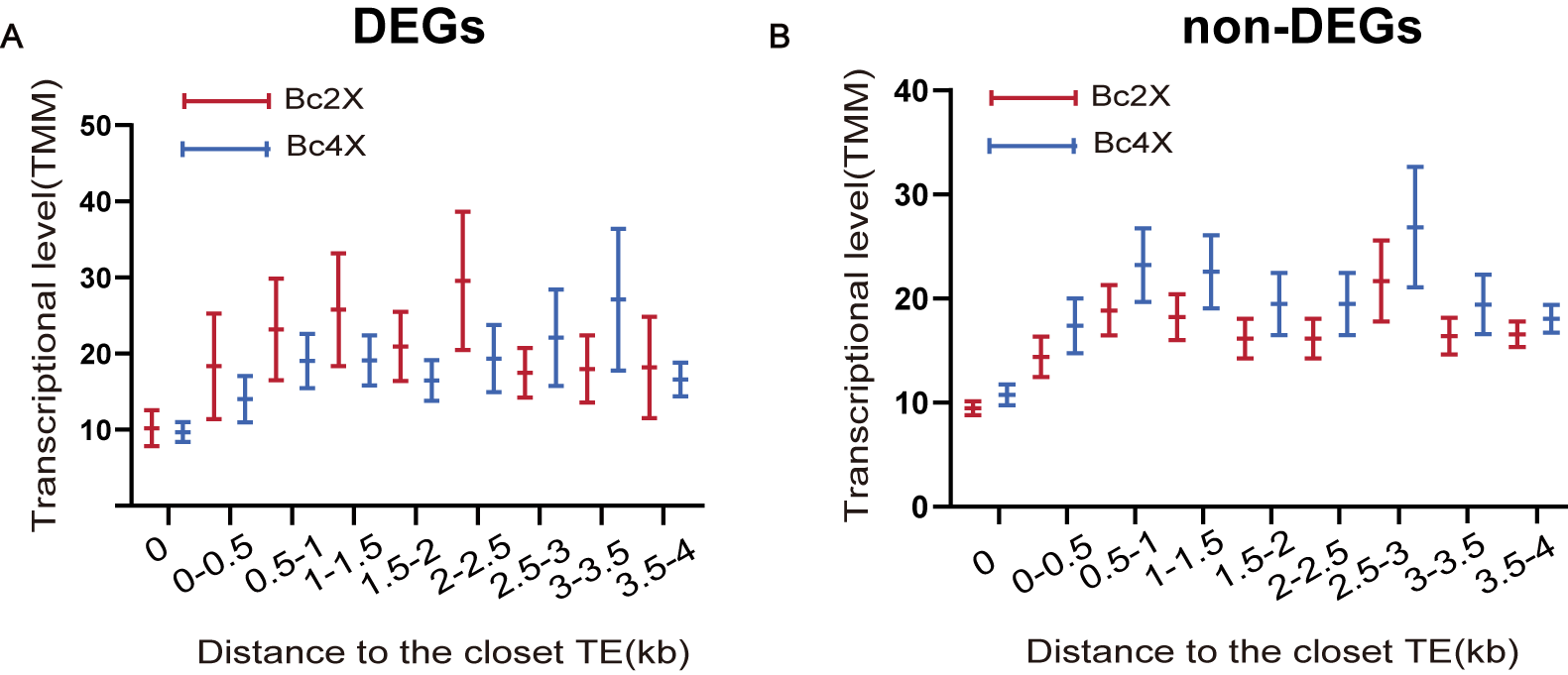

Supplement: Supplementary file 10 — Additional file 10. Figure S10. Transcription levels of genes related to the distance to the closest TE in Bc2X and Bc4X. (A) Transcription levels of DEGs related to the distance to the closest TE in Bc2X and Bc4X. “0” indicates genes overlapping with TEs in gene bodies. The error bars indicate the SEMs [Pearson’s r (Bc2X) = 0.053, P value = 0.003; r (Bc4X) = 0.017, P value = 0.004]. (B) Transcription levels of non-DEGs related to the distance to the closest TE in Bc2X and Bc4X. “0” indicates genes overlapping with TEs in gene bodies. The error bars indicate SEMs [Pearson’s r (Bc2X) = 0.016, P value = 0.005; r (Bc4X) = 0.015, P value = 0.01]. [file 43897_2025_145_MOESM10_ESM.tif]
